# An *In Vitro* Expansion System for Generation of Human iPS Cell-Derived Hepatic Progenitor-Like Cells Exhibiting a Bipotent Differentiation Potential

**DOI:** 10.1371/journal.pone.0067541

**Published:** 2013-07-25

**Authors:** Ayaka Yanagida, Keiichi Ito, Hiromi Chikada, Hiromitsu Nakauchi, Akihide Kamiya

**Affiliations:** 1 Division of Stem Cell Therapy, Center for Stem Cell Biology and Regenerative Medicine, The Institute of Medical Science, The University of Tokyo, Minato-ku, Tokyo, Japan; 2 Japan Science and Technology Agency, ERATO, Nakauchi Stem Cell and Organ Regeneration Project, Chiyoda-ku, Tokyo, Japan; 3 Institute of Innovative Science and Technology, Tokai University, Isehara, Kanagawa, Japan; University of Tampere, Finland

## Abstract

Hepatoblasts, hepatic stem/progenitor cells in liver development, have a high proliferative potential and the ability to differentiate into both hepatocytes and cholangiocytes. In regenerative medicine and drug screening for the treatment of severe liver diseases, human induced pluripotent stem (iPS) cell-derived mature functional hepatocytes are considered to be a potentially good cell source. However, induction of proliferation of these cells is difficult *ex vivo*. To circumvent this problem, we generated hepatic progenitor-like cells from human iPS cells using serial cytokine treatments *in vitro*. Highly proliferative hepatic progenitor-like cells were purified by fluorescence-activated cell sorting using antibodies against CD13 and CD133 that are known cell surface markers of hepatic stem/progenitor cells in fetal and adult mouse livers. When the purified CD13^high^CD133^+^ cells were cultured at a low density with feeder cells in the presence of suitable growth factors and signaling inhibitors (ALK inhibitor A-83-01 and ROCK inhibitor Y-27632), individual cells gave rise to relatively large colonies. These colonies consisted of two types of cells expressing hepatocytic marker genes (hepatocyte nuclear factor 4α and α-fetoprotein) and a cholangiocytic marker gene (cytokeratin 7), and continued to proliferate over long periods of time. In a spheroid formation assay, these cells were found to express genes required for mature liver function, such as cytochrome P450 enzymes, and secrete albumin. When these cells were cultured in a suitable extracellular matrix gel, they eventually formed a cholangiocytic cyst-like structure with epithelial polarity, suggesting that human iPS cell-derived hepatic progenitor-like cells have a bipotent differentiation ability. Collectively these data indicate that this novel procedure using an *in vitro* expansion system is useful for not only liver regeneration but also for the determination of molecular mechanisms that regulate liver development.

## Introduction

The liver is the largest internal organ in mammals and plays an important role in metabolism. It also performs various functions including glycogen storage, decomposition of red blood cells, plasma protein synthesis, and detoxification. Because of these many functions, it is difficult to construct an artificial liver replacement. Liver transplantation is considered the only effective treatment for end-stage liver diseases. However, it is limited by the shortage of suitable donor organs, the risk of rejection, infections, and lifelong immunosuppression. Although human embryonic stem (ES) cells derived from the inner cell mass of blastocysts maintain self-renewal and pluripotency [1], their use in clinical trials is limited because of the ethical concerns associated with human ES cell research. Human induced pluripotent stem (iPS) cells generated by reprogramming of somatic cells with four transcription factors (Oct3/4, Klf4, Sox2, and c-Myc) have similar properties to those of human ES cells [2]. Therefore, generation of hepatic cells using iPS technology may be beneficial for the treatment of severe liver diseases, screening of drug toxicities, and basic research of several hepatocytic disorders.

Liver organogenesis begins at early embryonic stages from the foregut endoderm. Endodermal cells are known to receive inductive signals from the septum transversum mesenchyme and adjacent cardiac region, namely bone morphogenetic protein (BMP) and fibroblast growth factor (FGF) [3], [4], [5], [6]. Subsequently, these cells commit to hepatoblasts that proliferate and migrate into the septum transversum to form the liver bud. Hepatoblasts are considered to be somatic stem/progenitor cells in fetal livers because they have a high proliferative potential and the ability to differentiate into both hepatocytes and cholangiocytes during the middle to late embryonic stages. Proliferation and differentiation of hepatoblast are regulated by several soluble factors. For example, hepatocyte growth factor (HGF), a mitogen of both hepatoblasts and mature hepatocytes, is important for expansion of the liver bud [7]. Likewise, oncostatin M (OSM) is a maturation inducer of fetal hepatic cells in the presence of glucocorticoid [8], [9]. Differentiation from human ES and iPS cells toward mature hepatocyte-like cells is induced by sequential addition of cytokines or transfection of genes involved in embryonic liver organogenesis [10], [11], [12], [13]. However, it is still difficult to obtain large numbers of highly functional hepatocytes from human iPS cells. In this regard, differentiation from human iPS cells toward highly proliferative hepatic progenitor cells will provide a method to obtain large quantities of hepatocytic cells.

Because differentiation from iPS cells toward hepatic lineage cells mimics *in vivo* step-wise developmental processes, human iPS cell-derived hepatic progenitor-like cells (HPCs) might exist at an appropriate time point during similar *in vitro* differentiation steps. Endodermal progenitor cells were established from human pluripotent cells, and these cells can differentiate into several endodermal lineage cells, such as pancreatic β cells, hepatocytes, and intestinal epithelial cells [14]. It has been recently shown that hepatic progenitor cells can be isolated from differentiated human ES cells using the cell surface marker N-cadherin [15]. However, methods for effective purification and cultivation of human iPS-derived HPCs have not been well established. We previously found that CD13 and CD133 are mouse hepatoblast-specific cell surface markers during the early and middle (E 9.5–14.5) stages of fetal development [16], [17]. Mouse CD13^+^CD133^+^ liver cells in the middle stage of fetal development express hepatic genes and differentiate into hepatocytic cells and cholangiocytic cells *in vitro* and *in vivo*. However, it remains unknown whether these mouse hepatoblast-specific markers are common to human hepatic progenitor cells. In addition, we previously reported that mouse embryonic fibroblasts (MEFs) have the ability to support *in vitro* proliferation of mouse hepatoblasts and hepatic gene expression [17]. In this study, because MEFs can be substituted for non-parenchymal cells in the liver, human iPS cell-derived HPCs were co-cultured with MEFs. Taken together, our data demonstrate that HPCs from human iPS cells can be highly purified using cell surface markers CD13 and CD133. Further investigation revealed that human iPS cell-derived HPCs exhibit a long-term proliferative potential and maintain bipotent differentiation toward hepatocytic cells and cholangiocytic cells.

## Materials and Methods

### Human iPS and ES cells and other-types of cells

A human iPS cell line, TkDA3-4, was established from human dermal fibroblasts (Cell Applications, Inc., San Diego, CA) as described previously [18]. Human ES cells (KhES-3) were obtained from the Institute for Frontier Medical Sciences, Kyoto University (Kyoto, Japan), with approval for human ES cell use granted by the Ministry of Education, Culture, Sports, Science, and Technology of Japan [19]. The Review Board of the Institute of Medical Science, The University of Tokyo, approved this research. The entire study was conducted in accordance with the Declaration of Helsinki. Human ES cells and human iPS cells were maintained according to previously described standard methods [18]. Briefly, they were cultured on irradiated mouse embryonic fibroblasts in Dulbecco's modified Eagle's medium/F-12 medium (DMEM/F-12; Sigma, St. Louis, MO) supplemented with 0.1 mM nonessential amino acids (Invitrogen, Carlsbad, CA), 1× penicillin streptomycin glutamine (Sigma), 20% knockout serum replacement (Invitrogen), 0.1 mM 2-mercaptoethanol (Invitrogen), and 5 ng/ml recombinant human basic fibroblast growth factor (basic FGF; Wako Pure Chem., Osaka, Japan). These cells were maintained on mitomycin C (Wako Pure Chem.)-treated MEFs. ES and iPS cells were passaged every 5 days to maintain them in an undifferentiated state.

HepG2 (RCB1886) cells were provided by RIKEN BRC through the National Bio-Resource Project of the MEXT, Japan. Cryopreserved human hepatocytes (Lot HEP187242; BioPredic International, Rennes, France) were used.

### Preparation of MEFs

E13.5 ICR or C57BL/6-Tg (CAG-EGFP) mouse embryos (Nihon SLC, Shizuoka, Japan) were dissected, and the head and internal organs were completely removed. The torso was minced and dissociated in 0.05% trypsin-EDTA (Sigma) for 30 min. After washing with phosphate-buffered saline (PBS; Sigma), cells were expanded in DMEM (Sigma) supplemented with 10% fetal bovine serum (FBS; Invitrogen) and 1× penicillin streptomycin glutamine. MEFs were treated with mitomycin C at 37°C for 2 h and used as feeder cells. Animal experiments were performed with approval of the Institutional Animal Care and Use Committee of the Institute of Medical Science, the University of Tokyo (permit number: PA09-21) and the Institutional Animal Care and Use Committee of Tokai University (permit number: 122047).

### Differentiation from human iPS cells toward the hepatic lineage and isolation of HPCs

For hepatic lineage cell differentiation, semi-confluent human iPS cells were cultured in RPMI 1640 (Sigma) containing 2% B27 supplement (Invitrogen). Cells were stimulated with 100 ng/ml recombinant human activin A (PeproTech, Rocky, NJ) at day 0–4, 10 ng/ml basic FGF and 20 ng/ml recombinant human BMP-4 (PeproTech) at day 5–8, and 40 ng/ml recombinant human HGF (PeproTech) at day 9–12. Cells were cultured at 37°C in a 10% O_2_ incubator at day 0–4 and in 5% O_2_ at day 5–12.

HPCs were isolated from human iPS cells stimulated with cytokines. After 12 days of culture, cells were trypsinized using 0.05% trypsin-EDTA (Sigma). Trypsinized cells were washed with PBS containing 3% FBS, and then incubated with antibodies against cell surface proteins (shown in **Table S1**) for 1 h at 4°C. After washing with PBS containing 3% FBS and staining the dead cells with propidium iodide, the cells were analyzed and sorted using a MoFlo™ fluorescence-activated cell sorter (Dako, Glostrup, Denmark) and a FACSAria cell sorter (Becton Dickinson, Franklin Lakes, NJ).

### HPC culture and passaging

Mitomycin C-treated MEFs (2×10^5^ cells/well) were plated onto 0.1% gelatin (derived from porcine skin; Sigma)-coated 12-well plates the day before sorting. CD13^high^CD133^+^ cells were sorted onto MEF feeder layers plated at a low density (280 cells per cm^2^). The standard culture medium was a 1∶1 mixture of H-CFU-C medium and DMEM. H-CFU-C medium consisted of DMEM/F-12 supplemented with 10% FBS (Nichirei Biosciences, Tokyo, Japan), 1× Insulin-Transferrin-Selenium X (Invitrogen), 10 mM nicotinamide (Sigma), 10^−7^ M dexamethasone (Sigma), 2.5 mM HEPES buffer solution (Invitrogen), 1× penicillin streptomycin glutamine, and 1× nonessential amino acids. For expansion, the cells were cultured in standard medium supplemented with 0.25 µM A-83-01 (Tocris Bioscience, Bristol, United Kingdom), 10 µM Y-27632 (Wako Pure Chem.), 40 ng/ml HGF, and 20 ng/ml recombinant human epidermal growth factor (EGF; PeproTech). The medium was replaced every 3 days. After 10–12 days of culture, colonies formed by individual human iPS cell-derived CD13^high^CD133^+^ cells were trypsinized using 0.05% trypsin-EDTA and replated onto freshly plated MEFs.

### Isolation of secondary CD13^+^ and/or CD133^+^ cells from human iPS cell-derived HPCs

After differentiation of human iPS cells induced by cytokines, CD13^high^CD133^+^ cells were cultured on MEFs derived from C57BL/6-Tg (CAG-EGFP) mice. After 12 days of culture, HPC colonies were trypsinized and stained with PE- and APC-conjugated antibodies against human CD13 and CD133, respectively. CD13^+^ and/or CD133^+^ cells in the GFP-negative fraction were sorted using a MoFlo™ fluorescence-activated cell sorter. Sorted cells were seeded onto freshly plated MEFs, and the colony forming activities of these cells were analyzed.

### Immunocytochemistry

Cultured cells were washed with PBS and fixed with 4% paraformaldehyde/PBS. After three washes with PBS, cells were permeabilized with 0.25% Triton X-100 (Sigma)/PBS for 10 min, washed with PBS, and incubated with 5% donkey serum (Millipore, Bedford, MA) in PBS for 1 h at room temperature. The cells were then incubated with diluted primary antibodies either overnight at 4°C. Primary antibodies are listed in **Table S1**. The cells were washed with PBS several times, and then incubated with diluted secondary antibodies for 40 min at room temperature. Then, the cells were washed with PBS and their nuclei were stained with 4′,6-diamidino-2-phenylindole dihydrochloride (DAPI; Sigma). Normal goat IgG (Santa Cruz Biotechnology, Santa Cruz, CA), negative control mouse IgG1 (Dako), and normal rabbit IgG (Santa Cruz Biotechnology) were used as negative controls for the appropriate antibodies. Colonies were imaged under a Carl Zeiss Axio Observer Z1 using AxioVision version 4.8 software (Carl Zeiss, Jena, Germany). α-fetoprotein (AFP)- and hepatocyte nuclear factor 4α (HNF4α)-positive colonies were counted using an ArrayScan VTI HCS Reader (Thermo Scientific, Waltham, MA).

### Induction of mature hepatocytic functional genes by spheroid formation

Colonies derived from CD13^high^CD133^+^ cells were passaged and trypsinized with 0.05% trypsin-EDTA, washed in DMEM containing 10% FBS, and counted. The culture medium for spheroid formation was DMEM supplemented with 10% FBS, 1× nonessential amino acids, 1× penicillin streptomycin glutamine, and 10^−7^ M dexamethasone, with or without 20 ng/ml recombinant human OSM (R&D Systems, Minneapolis, MN). Individual drops (40 µl) containing 1×10^4^ cells were plated on the inside of lids of 100-mm dishes containing PBS (to avoid desiccation). After 3 days of culture, the spheroids were collected and analyzed.

### Real-time RT-PCR analysis

For analysis of hepatic functional gene expressions, total RNA was extracted from human iPS cells, colonies derived from CD13^high^CD133^+^ cells, and spheroids using TRIzol (Invitrogen). First-strand cDNA synthesized using a High Capacity cDNA Reverse Transcription Kit (Life Technologies, Carlsbad, CA) was used as a template for PCR amplification. For semiquantitative RT-PCR, cDNA samples were normalized by the number of hypoxanthine phosphoribosyltransferase 1 (HPRT1) mRNA copies. The Universal Library (Roche Diagnostics, Basel, Switzerland) was used for quantitative RT-PCR assays of AFP, catechol-O-methyltransferase (COMT), chemokine (C-X-C motif) receptor 4 (CXCR4), cytochrome P450 (CYP) 3A4, CYP3A7, CYP7A1, epoxide hydrolase 1, microsomal (xenobiotic) (EPHX1), flavin containing monooxygenase 5 (FMO5), goosecoid homeobox (GSC), hematopoietically expressed homeobox (hHex), hepatocyte nuclear factor (HNF) 3β, HNF4α, HPRT1, monoamine oxidase A (MAOA), MAOB, Mix paired-like homeobox (MIXL1), one cut homeobox 1 (ONECUT1), Sox17, sulfotransferase family, cytosolic, 1A, phenol-preferring, member 1 (SULT1A1), SULT1A2, and T transcripts. The primer sequences and probe numbers for each gene are shown in Table S2.

### Induction of cholangiocytic cyst formation by human iPS cell-derived CD13^high^CD133^+^ cells

Colonies derived from CD13^high^CD133^+^ cells were passaged and trypsinized using 0.05% trypsin-EDTA, washed in DMEM containing 10% FBS, and then counted. The cells were then combined with an extracellular matrix gel consisting of a mixture of 40% collagen type-I (Nitta Gelatin, Osaka, Japan) and 40% Matrigel (BD Biosciences, Bedford, MA), and cultured in 24-well culture plates (1500 cells/50 µl extracellular matrix gel/well). After the 10 min incubation, culture medium was added, followed by incubation for 10–12 days with medium changes every 3 days. The culture medium was a 1∶1 mixture of H-CFU-C medium and DMEM/F-12 supplemented with 2% B27 supplement, 0.25 µM A-83-01, 10 µM Y-27632, 20 ng/ml EGF, 40 ng/ml HGF, 40 ng/ml recombinant human Wnt-3a (R&D Systems), and 100 ng/ml recombinant human R-spondin 1 (PeproTech). Cysts in gels were stained according to previously described methods [20], and analyzed under a LSM700 confocal microscope (Carl Zeiss). The antibodies are listed in Table S1.

### Albumin secretion assay

To detect human albumin secretion, colonies from human iPS cell-derived HPCs (3rd culture) were cultured for a long time (19 days). These cells were differentiated by cell-cell interactions. The differentiated cells and HepG2 cells (standard control) were incubated for 3 days in hepatocyte differentiation medium. The hepatocyte differentiation medium was DMEM supplemented with 10% FBS, 1× nonessential amino acids, 1× penicillin streptomycin glutamine, 10^−7^ M dexamethasone, and 1% dimethyl sulfoxide (Sigma). Conditioned medium was saved and debris was removed by centrifugation. Human albumin was detected using a Human Albumin ELISA Quantitation Set (Bethyl Laboratories, Montgomery, TX) according to the manufacturer's protocol.

### Statistics

Calculations of statistically significant differences between samples using the Student's two-tailed t test as well as the standard deviation (SD) were performed using Microsoft Excel 2007 software.

## Results

### Differentiation from human iPS cells toward hepatic lineage cells

**Figure 1:**
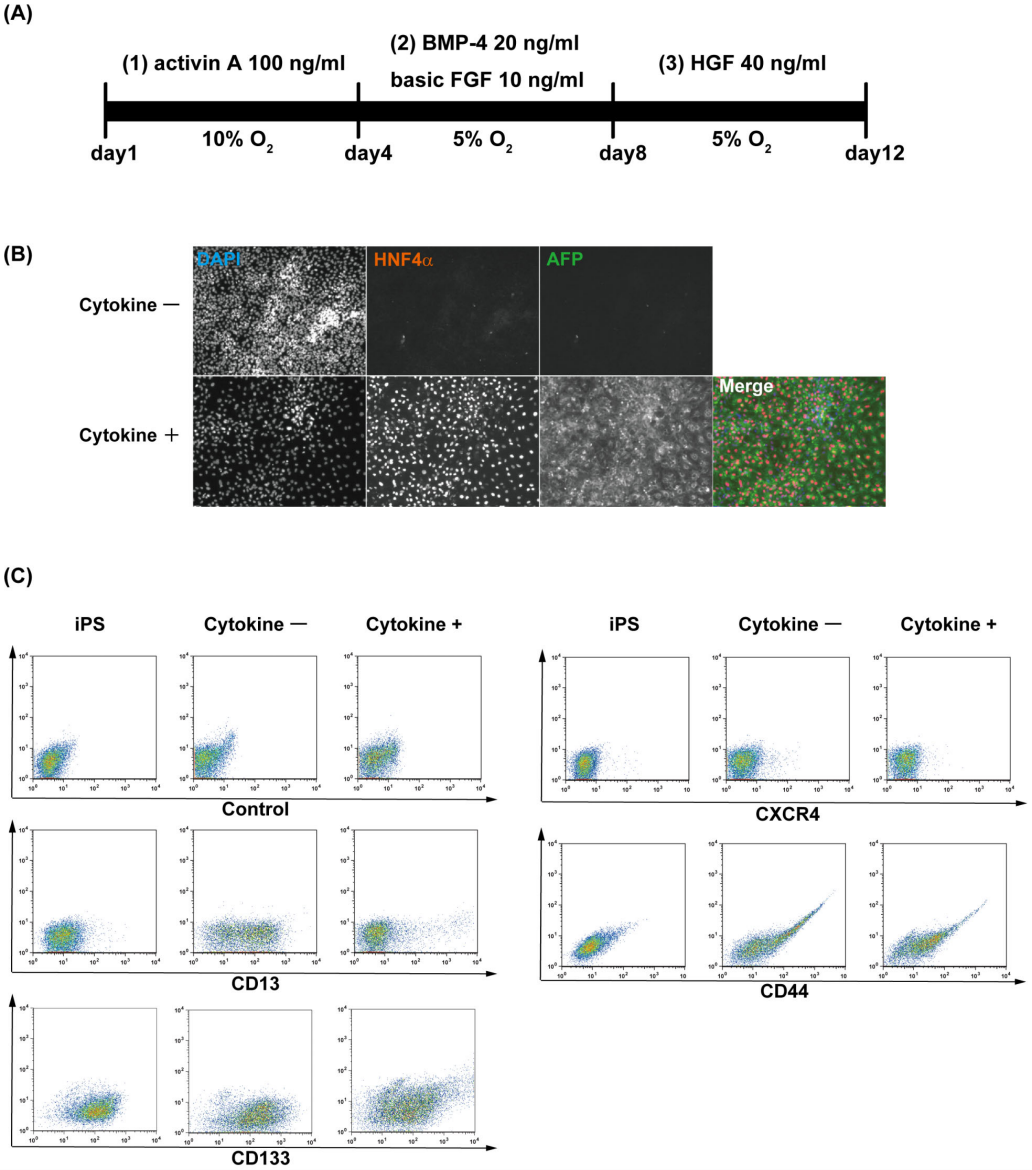
Differentiation from human iPS cells toward hepatic lineage cells. (**A**) Schematic of the experimental procedure. Human iPS cells were sequentially stimulated with various cytokines: (1) activin A, (2) basic FGF and BMP-4, and (3) HGF. The cells were cultured in 10% O_2_ for days 0–4 and 5% O_2_ for days 5–12. (**B**) After 12 days of culture with or without cytokines, cells were stained with antibodies against AFP and HNF4α. Nuclei were counterstained with DAPI. (**C**) Expression of cell surface markers in human iPS cell-derived hepatic lineage cells.

Human ES cells and iPS cells have been reported to differentiate into hepatocytes using a stage-wise process that mimics developmental processes [10], [11]. We speculated that human iPS cell-derived hepatic lineage cell cultures contained HPCs induced by several cytokines such as activin A, basic FGF, BMP-4, and HGF. For hepatic lineage cell differentiation, TkDA3-4 human iPS cells were seeded onto mitomycin C-treated MEFs and stimulated with the cytokines shown in **Figure 1A**. After 12 days of culture, expression of fetal hepatocytic markers (AFP and HNF4α) were observed in cells stimulated with cytokines (**Figure 1B**). We then tried to isolate HPCs from hepatic lineage cell cultures. As shown previously, several cell surface markers, such as CD34, CD44, CD49f, CD56, CD117, CD326, and CXCR4, have been reported to be expressed in mouse and human endodermal and hepatic progenitor cells, and fetal hepatic cells [21], [22], [23], [24], [25], [26]. We recently found that CD13 and CD133 are specific cell surface markers for mouse hepatoblasts [16], [17]. To characterize human iPS cell-derived hepatic lineage cells, undifferentiated human iPS cells and cells cultured with or without cytokines were analyzed with antibodies against hepatic progenitor cell-related surface markers by flow cytometry. CD13 was barely expressed in human iPS cells, but was induced during the 12 days of culture without cytokines, indicating that spontaneous differentiation of human iPS cells generated CD13^mid^ cells. Interestingly, CD13^high^ cells were only detected in cultures stimulated with cytokines and expressed CD133, another hepatoblast surface marker (**Figure 1C** and **Figure S1A**). Moreover, CD13^high^CD133^+^ cells expressed other hepatic cell surface markers, CD49f and CD326 (**Figure S1A**, and data not shown). In contrast, expression of CD34 and CD56 was barely detected in CD13^high^CD133^+^ cells (**Figure S1B**). Expression of CD44 and CXCR4 were not significantly changed by the addition of cytokines (**Figure 1C**).

### Expressions of differentiation marker genes during differentiation of human iPS cells

**Figure 2:**
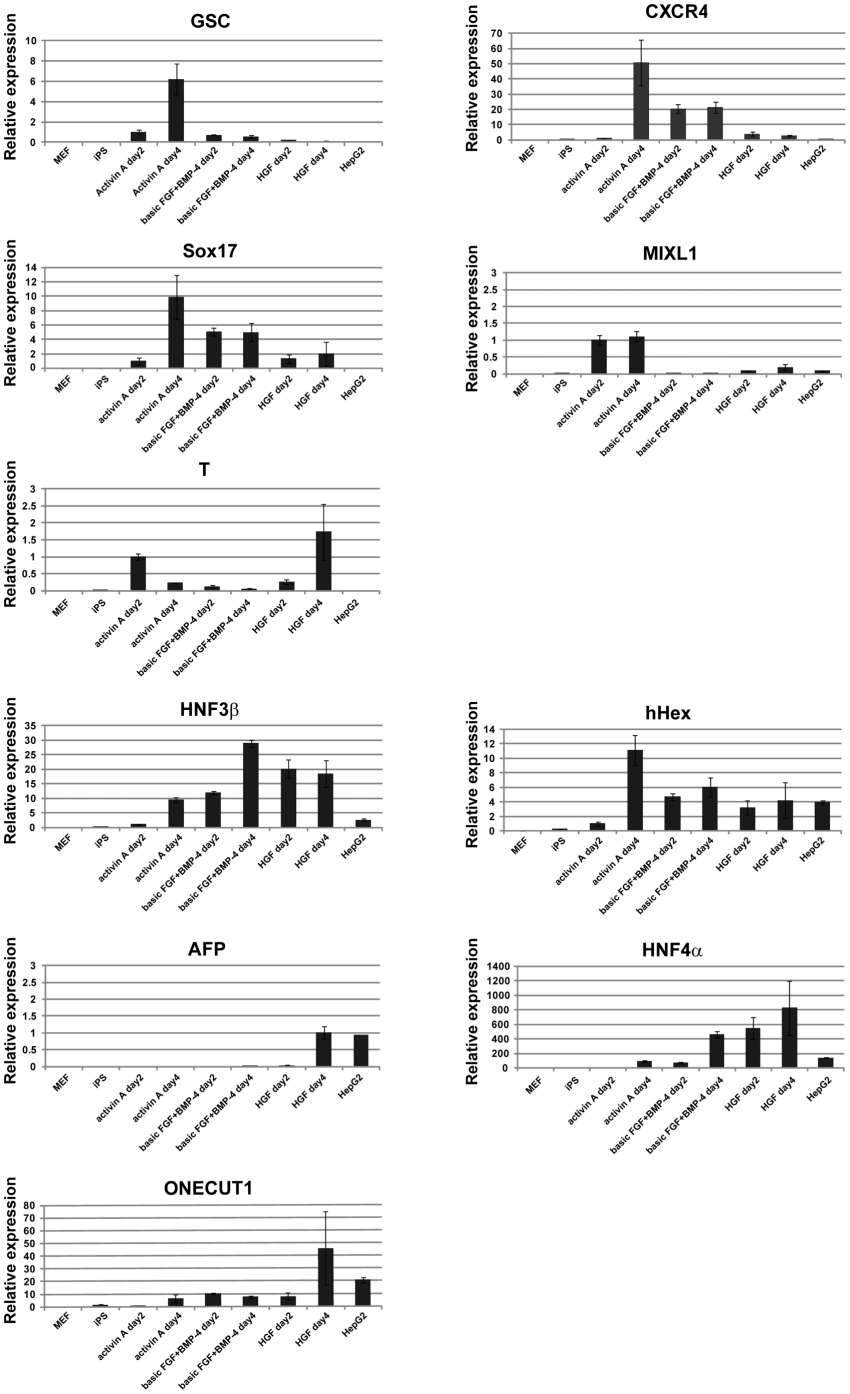
Expressions of undifferentiated and differentiated cell markers in human iPS cell-derived cells. The expressions of marker genes for mesodermal and endodermal cells (GSC, CXCR4, Sox17, MIXL1, T, HNF3β, and hHex) and hepatic cells (AFP, HNF4α, and ONECUT1) were examined in normal human iPS cells (iPS) and differentiated iPS cells. For quantitative PCR analyses, mRNAs were purified from MEF, HepG2 cells, normal iPS cells, and iPS cells stimulated with activin A on days 2 and 4, basic FGF and BMP-4 on days 2 and 4, and HGF on days 2 and 4. The results are represented as the mean colony counts ± SD (triplicate samples).

When differentiation of human pluripotent stem cells into endodermal cells was induced by addition of activin A, several differentiation marker genes were transiently upregulated [26]. We analyzed the expressions of mesodermal and endodermal marker genes (GSC, CXCR4, Sox17, MIXL1, T, HNF3β, and hHex) and hepatocytic marker genes (AFP, HNF4α, and ONECUT1) (**Figure 2**). The hepatocytic marker gene expressions were highly increased when iPS cells were stimulated by HGF. We found that most cells derived from human iPS cells differentiated into AFP^+^ and HNF4α^+^ hepatocytic cells during our differentiation steps, although several cell clusters that could not differentiate into the hepatic lineage remained (**Figure S2A**). The pluripotency maker Oct3/4 was not expressed in the AFP^+^ hepatocytic cells at the end of the differentiation steps (**Figure S2B**). Part of AFP^−^ undifferentiated cell clusters expressed T (**Figure S2C**), indicating that expression of T was detected by RT-PCR at the end of the differentiation. In contrast, the expressions of other mesodermal and endodermal marker genes (GSC, CXCR4, Sox17, and MIXL1) were transiently upregulated at the endodermal differentiation stage (by stimulation with activin A) and decreased during hepatocytic differentiation. These results suggested that the iPS cells differentiated into hepatocytic cells through the definitive endodermal pathway.

### Purification and *in vitro* culture of human iPS cell-derived CD13^high^CD133^+^ cells

**Figure 3:**
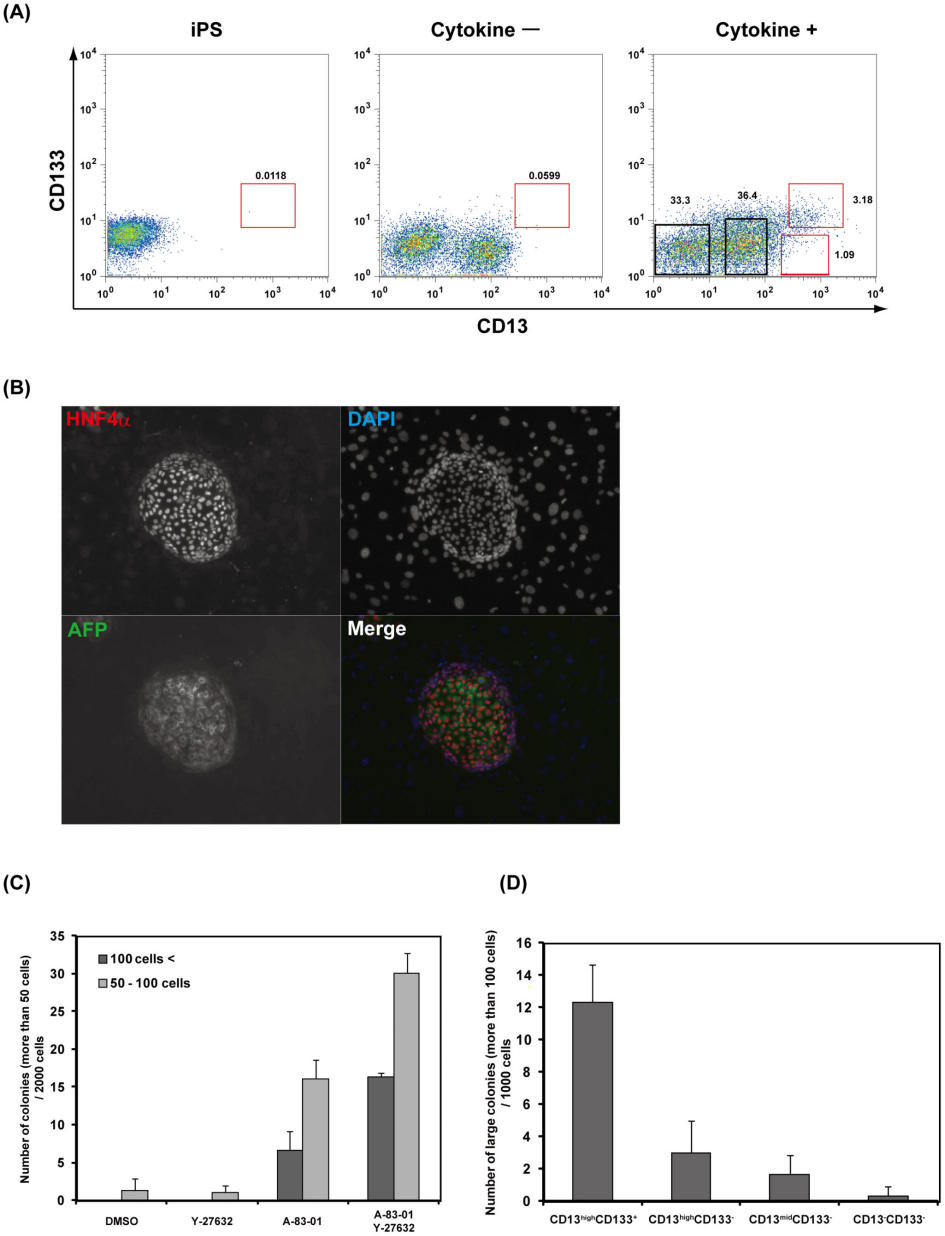
Isolation of HPCs from human iPS cell-derived hepatic lineage cells. (**A**) Expression of hepatic progenitor markers in undifferentiated human iPS cells and differentiated cells. After 12 days of culture with or without cytokines, cells were stained with antibodies against CD13 and CD133, and then analyzed by flow cytometry. Ratios of CD13^high^CD133^+^ cells are shown. (**B**) Representative images of a colony derived from a single CD13^high^CD133^+^ cell. Colonies were stained with antibodies against AFP and HNF4α. Nuclei were counterstained with DAPI. (**C**) Culture condition of the human iPS cell-derived hepatic progenitor colony assay. CD13^high^CD133^+^ cells were sorted and cultured on MEFs in the presence or absence of A-83-01 (ALK inhibitor) and Y-27632 (ROCK inhibitor). Results are represented as the mean colony count ± SD (triplicate samples). (**D**) CD13^−^CD133^−^, CD13 weakly single positive, CD13^mid^ single positive and CD13^high^CD133^+^ cells were sorted onto MEFs. The cells were cultured in standard culture media in the presence of A-83-01 and Y-27632. Large colonies (containing more than 100 cells) derived from individual sorted cells were counted. Results are represented as the mean colony count ± SD (triplicate samples).

Next, we analyzed the characteristics of human iPS cell-derived cells expressing hepatic progenitor cell surface markers, CD13 and CD133. During fetal liver development, hepatic progenitor cells interact with non-parenchymal cells such as mesenchymal cells, fibroblasts, mesothelial cells and endothelial cells [27], [28], [29], [30]. We recently established a co-culture system consisting of mouse early fetal hepatic progenitor cells and mesenchymal feeder cells [17]. Cell-cell interactions with mesenchymal cells are required for effective expansion of early fetal hepatic progenitor cells *in vitro*. Thus, we sorted CD13^high^CD133^+^ cells derived from human iPS cell cultures stimulated with cytokines (**Figure 3A**), and cultured the sorted cells on MEFs in H-CFU-C medium supplemented with EGF and HGF. After 12 days of culture, single CD13^high^CD133^+^ cells produced colonies expressing both AFP and HNF4α (**Figure 3B**). These results suggest that the CD13^high^CD133^+^ fraction contains HPCs. In this culture system, the addition of an ALK inhibitor, A-83-01, was required for effective expansion of CD13^high^CD133^+^ cells (**Figure 3C**). The combination of A-83-01 and Y-27632 significantly increased the number of large colonies derived from CD13^high^CD133^+^ cells. Based on these results, we used standard culture medium supplemented with EGF, HGF, A-83-01 and Y-27632 for expansion of human iPS cell-derived HPCs. To determine which fraction contained HPCs, we sorted the cultured cells into CD13^−^, CD13^mid^, CD13^high^CD133^−^, and CD13^high^CD133^+^ fractions, and the cells were plated individually onto MEFs. After these cells were cultured, large single cell-derived colonies containing over 100 cells were obtained and counted. As shown in **Figure 3D**, we found that the CD13^high^CD133^+^ fraction contained many HPCs and formed large colonies expressing AFP and HNF4α.

### Long-term *in vitro* expansion of human iPS cell-derived HPCs

**Figure 4:**
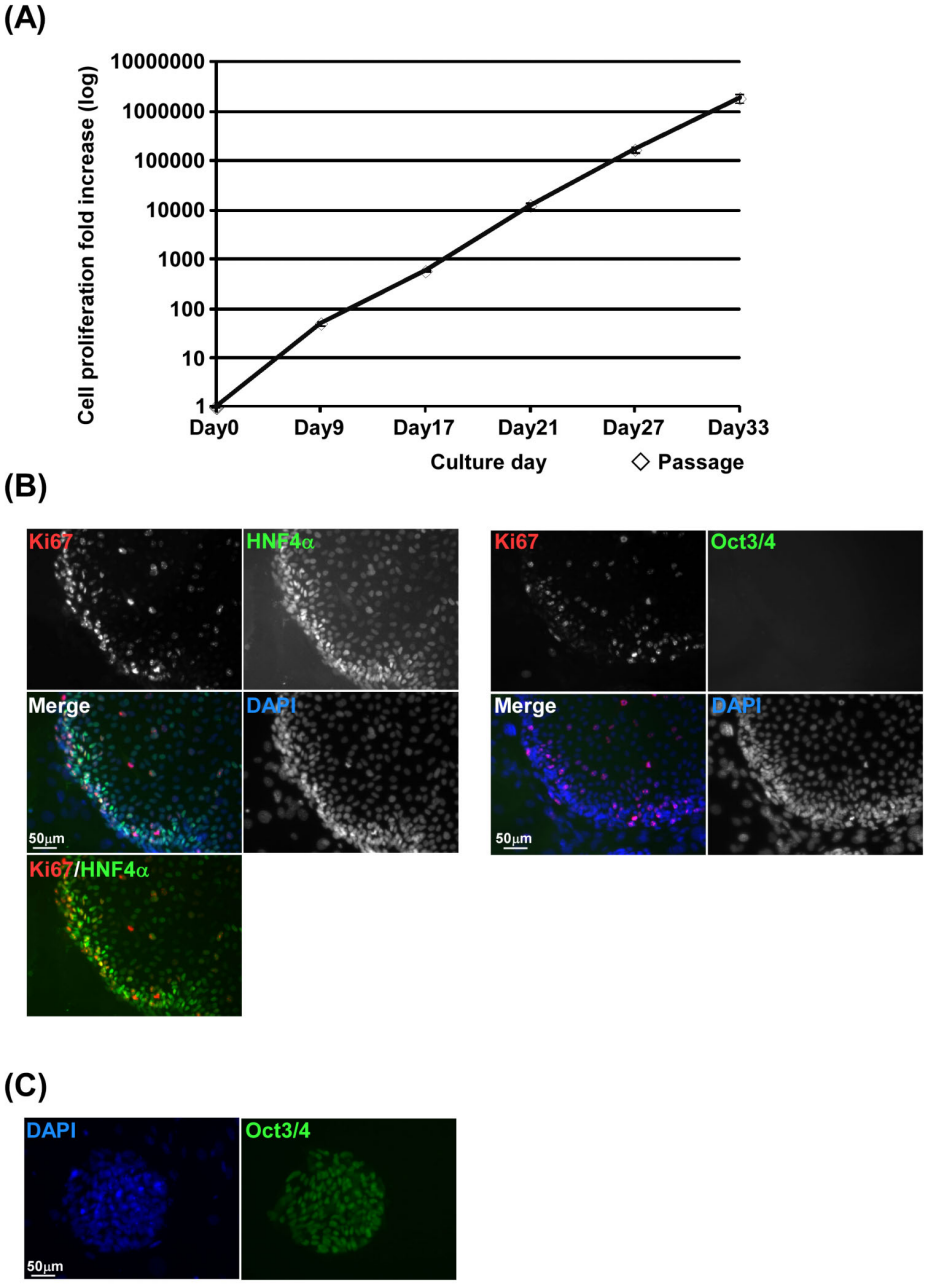
*In vitro* long-term expansion of CD13^high^CD133^+^ cells. (**A**) Colonies derived from CD13^high^CD133^+^ cells were trypsinized and replated onto MEFs. The number of cells was counted at each replating step. The cells continued to proliferate for more than 1 month. A representative growth curve is shown. Similar results were obtained in two independent experiments. (**B**) Expression of the proliferation marker Ki67 in human iPS cell-derived hepatocytic colonies. After the 4th passage, the colonies were stained with antibodies against Ki67, HNF4α, and Oct3/4. (**C**) Human iPS cells were stained with an antibody against Oct3/4.

**Figure 5:**
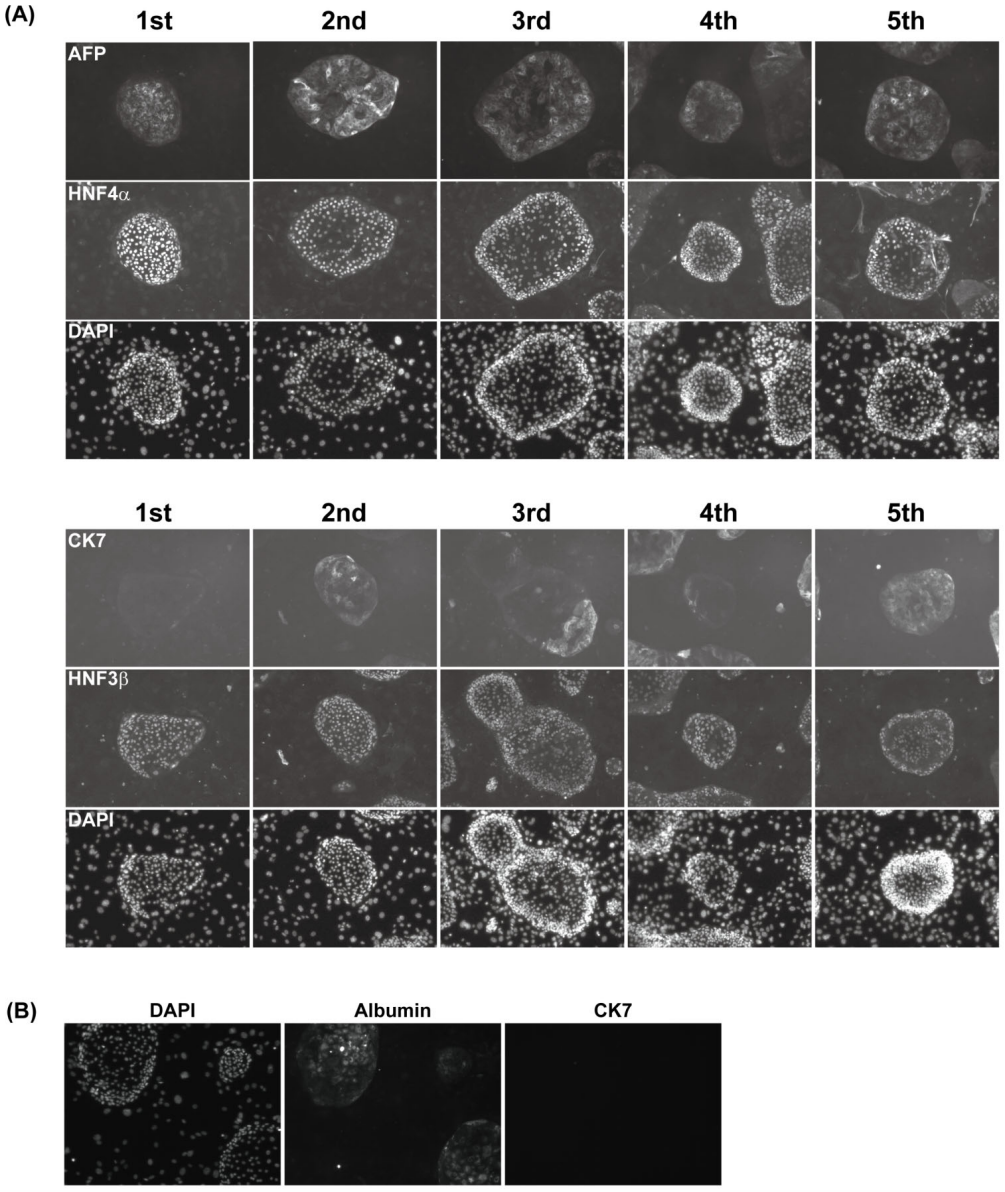
Expression of hepatocytic and cholangiocytic marker genes in human iPS cell-derived hepatic progenitor-like colonies. (**A**) Expression of hepatocytic and cholangiocytic markers during *in vitro* expansion. Colonies derived from CD13^high^CD133^+^ cells were trypsinized and replated onto MEFs. Cells were fixed at each replating step. An endodermal marker (HNF3β), hepatocytic markers (AFP and HNF4α) and a cholangiocytic marker (CK7) were stained with specific antibodies. Nuclei were counterstained with DAPI. (**B**) Expression of albumin in colonies derived from human iPS cell-derived HPCs. Albumin but not CK7 was detected in several colonies in the 1st culture.

One of the most important characteristics of hepatic stem/progenitor cells is a high proliferative ability. To analyze whether human iPS cell-derived HPCs could proliferate in long-term cultures *in vitro*, colonies derived from CD13^high^CD133^+^ cells were trypsinized and replated onto new feeder cells. The number of cells was counted at each replating step. The cells continued to proliferate for more than 1 month (**Figure 4A**). After the 4th passage, the HNF4α^+^ human iPS cell-derived HPC colonies expressed the proliferation marker Ki67 (**Figure 4B**). In contrast, these HPC colonies did not express the pluripotency marker Oct3/4, which is expressed in human iPS cells (**Figure 4B and C**). Next, we analyzed the expression of hepatocytic and cholangiocytic marker genes in human iPS cell-derived HPCs at each replating step. Colonies were stained with specific antibodies against AFP, HNF3β, HNF4α and cytokeratin 7 (CK7). Expression of the endodermal marker, HNF3β, and hepatocytic markers, AFP and HNF4α, was observed in primary colonies and maintained during *in vitro* expansion (**Figure 5A**). In particular, human iPS cell-derived HPCs were able to proliferate for more than 3 months and still expressed the hepatocytic markers HNF4α and AFP (**Figure S3A and B**). Cholangiocytic marker CK7 was not expressed in primary colonies but was induced after several passages. A small number of cells in colonies of the 1st culture expressed albumin, a mature hepatocytic marker gene (**Figure 5B**). CD13^high^CD133^+^ cells derived from human iPS cells could expand *in vitro* over a long term while maintaining hepatocytic and cholangiocytic marker gene expression, indicating that these cells have hepatic progenitor-like potentials.

**Figure 6:**
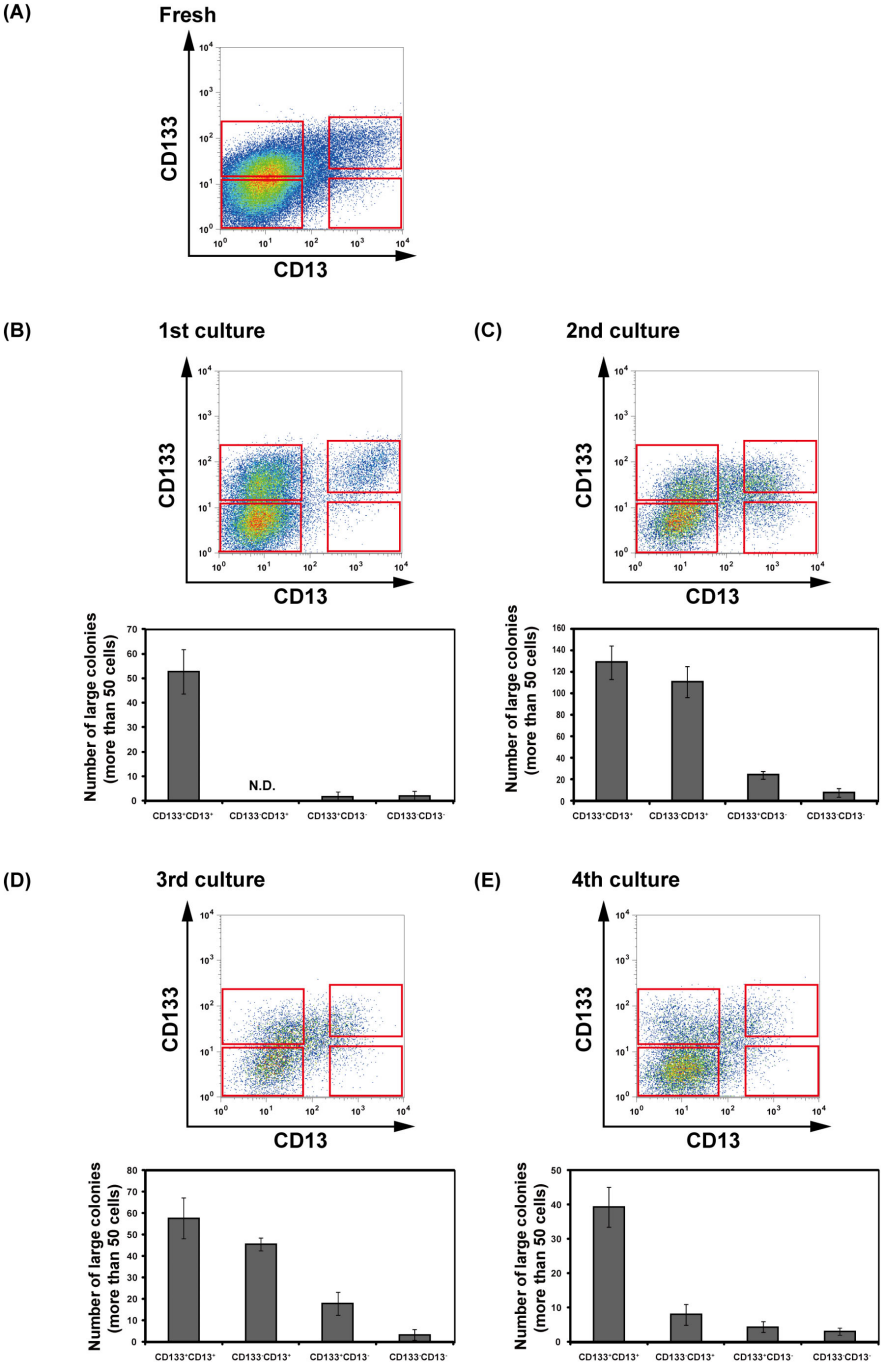
Changes of HPC surface marker expression in human iPS cell-derived hepatic progenitor-like colonies. (**A**) After 12 days of culture with cytokines, CD13^high^CD133^+^ cells were sorted onto GFP-MEFs. After 10–12 days, cells were trypsinized and stained with antibodies against CD13 and CD133. Expression of CD13 and CD133 was analyzed by flow cytometry. (**B–E**) Colony forming activity of CD13^+^CD133^+^, CD13^+^CD133^−^, CD13^−^CD133^+^, and CD13^−^CD133^−^ fractions of human iPS cell-derived HPCs. At every replating step, cells stained with antibodies against CD13 and CD133 were sorted onto new GFP-MEFs, and their colony-forming activity was analyzed. Results are represented as the mean colony count ± SD (duplicate samples). N.D. shows “not determined”.

As shown above, CD13^high^CD133^+^ cells spontaneously differentiated into CK7-positive cholangiocyte-like cells AFP and HNF4α-negative non-hepatocytic cells during passaging. To determine the cell fraction containing self-renewing cells after serial passaging, human iPS cell-derived HPC colonies at each replating step were analyzed using antibodies against CD13 and CD133 (**Figure 6**). In addition to the CD13^+^CD133^+^ fraction, CD13^−^CD133^+^, CD13^+^CD133^−^ and CD13^−^CD133^−^ fractions were found in cultures derived from human iPS cell-derived CD13^high^CD133^+^ cells, indicating that HPCs spontaneously differentiated and lost expression of CD13 and CD133. We analyzed the colony-forming activities of these fractions at replating steps. Cells at each replating step were sorted and cultured on MEFs for 12 days, and then HNF4α-positive colonies derived from each fraction were counted (**Figure 6B–D**). The rate of HNF4α positive colony formation was high in cultures derived from CD13^+^CD133^+^ cells at each replating step. Interestingly, CD13^+^CD133^−^ cells after several passages also formed many HNF4α-positive colonies. These results suggest that CD13 is likely to be a useful surface marker to purify self-renewing HPCs after serial passaging. Next, we analyzed whether the CD13^+^ cells among human iPS cell-derived HPCs had self-renewal potency. When human iPS cell-derived HPCs were passaged twice, the 3rd cultured-cells were stained with suitable antibodies, and CD13^+^ and CD13^−^ cells were sorted. After 11 days of culture, the expanded colonies (4th culture) derived from CD13^+^ and CD13^−^ cells were analyzed by flow cytometry (**Figure S3C**). Interestingly, the HPC colonies derived from CD13^+^ cells contained many CD13^+^ cells. In contrast, the HPC colonies derived from CD13^−^ cells barely contained CD13^+^ cells. We obtained similar results using the 5th cultured-cells. In addition, we compared the colony-forming abilities of CD13^+^ and CD13^−^ cells in the 5th culture. As shown in **Figure S3D**, CD13^+^ cells were able to form colonies more efficiently than CD13^−^ cells. These results suggested that CD13^+^ cells maintained the self-renewal-like ability to proliferate for a long time.

### Differentiation of human iPS cell-derived HPCs toward both mature hepatocytic and cholangiocytic cells

**Figure 7:**
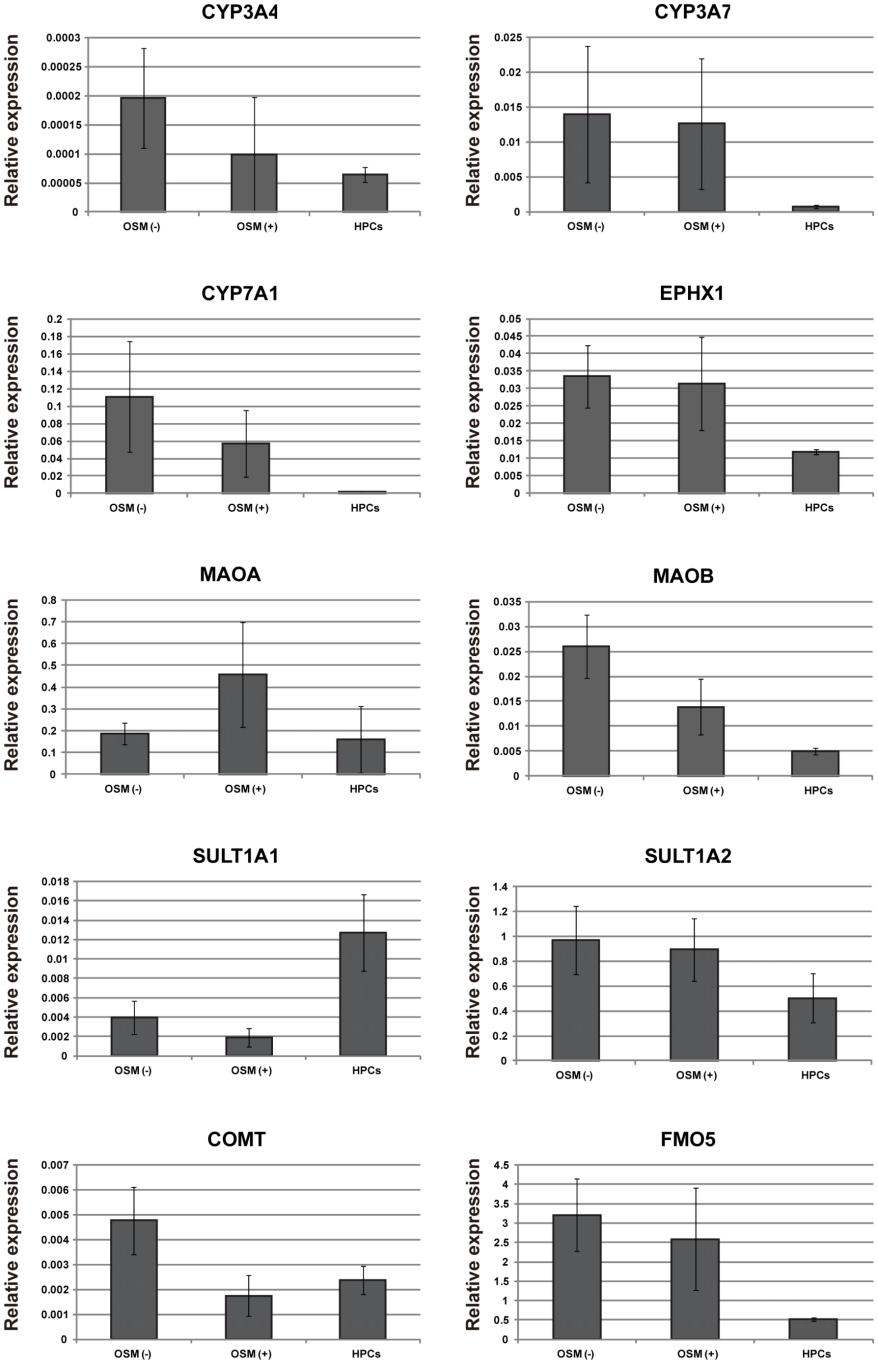
Expressions of hepatic functional genes in differentiated HPCs. The levels of mRNAs encoding phase 1 and 2 enzymes in human iPS cell-derived HPCs from the 3rd culture, and spheroids derived from human iPS cell-derived HPCs from the 3rd culture are shown as the fold values relative to the levels in uncultured human hepatocytes. Spheroid formation was induced by hanging drop culture in the presence or absence of OSM. The results are represented as the mean colony counts ± SD (spheroid culture, n = 6; HPCs, n = 3; uncultured human hepatocytes, n = 2).

Hepatic progenitor cells in fetal liver development have a bipotent differentiation ability toward hepatocytes and cholangiocytes [23], [31]. Therefore, we assessed whether human iPS cell-derived HPCs had the potential to differentiate into mature hepatocytic cells. To maintain and induce the functions of mature hepatocytes *in vitro*, three-dimensional (3D) biological structures are known to be important [32], [33], [34]. For this purpose, the hanging drop method is often used to self-assemble hepatic stem/progenitor cells into 3D aggregates (spheroids). For human iPS cell-derived hepatocytic differentiation, human iPS cell-derived HPCs in the 3rd culture were trypsinized and suspended in hepatocyte medium supplemented with dexamethasone, with or without OSM, for spheroid formation (**Figure S4A**). After 3 days of spheroid culture, the expressions of hepatic functional genes in human iPS-derived cells were compared with those in primary human hepatocytes (**Figure 7**). Both phase 1 enzymes (CYP3A4, CYP3A7, CYP7A1, EPHX1, FMO5, and MAOB) and phase 2 enzymes (COMT and SULT1A2) were expressed in spheroids derived from human iPS cell-derived HPCs from the 3rd culture. Except for MAOA, the expression levels of mature hepatocyte functional marker genes in spheroids without OSM were higher than those in spheroids with OSM. The expressions of several functional genes in human iPS-derived spheroids were significantly lower than those in primary hepatocytes, whereas mature hepatocyte functional marker genes, FMO5, MAOA, MAOB, and SULT1A2, were expressed highly or at similar levels compared with a previous report on human ES and iPS cell-derived hepatocytic cells [12].

Albumin secretion is one of the most important functions of mature hepatocytes. We cultured human iPS cell-derived HPCs at a high density for a long time and induced hepatic maturation by cell-cell interactions [35]. The HPCs in the high-density culture exhibited significant expression of albumin (**Figure S4B**). Subsequently, the amounts of albumin protein in the conditioned culture media were analyzed by enzyme-linked immunosorbent assays. We compared the albumin secretion levels in these HPCs with those in HepG2 cells. As shown in **Figure S4C**, the albumin secretion levels of HPCs were higher than those of HepG2 cells, and the secreted protein levels were similar to those in a previous report on human iPS cell-derived hepatocytic cells [12]. These results suggest that human iPS cell-derived HPCs have the potential to differentiate into mature hepatocytic cells after *in vitro* expansion.

**Figure 8:**
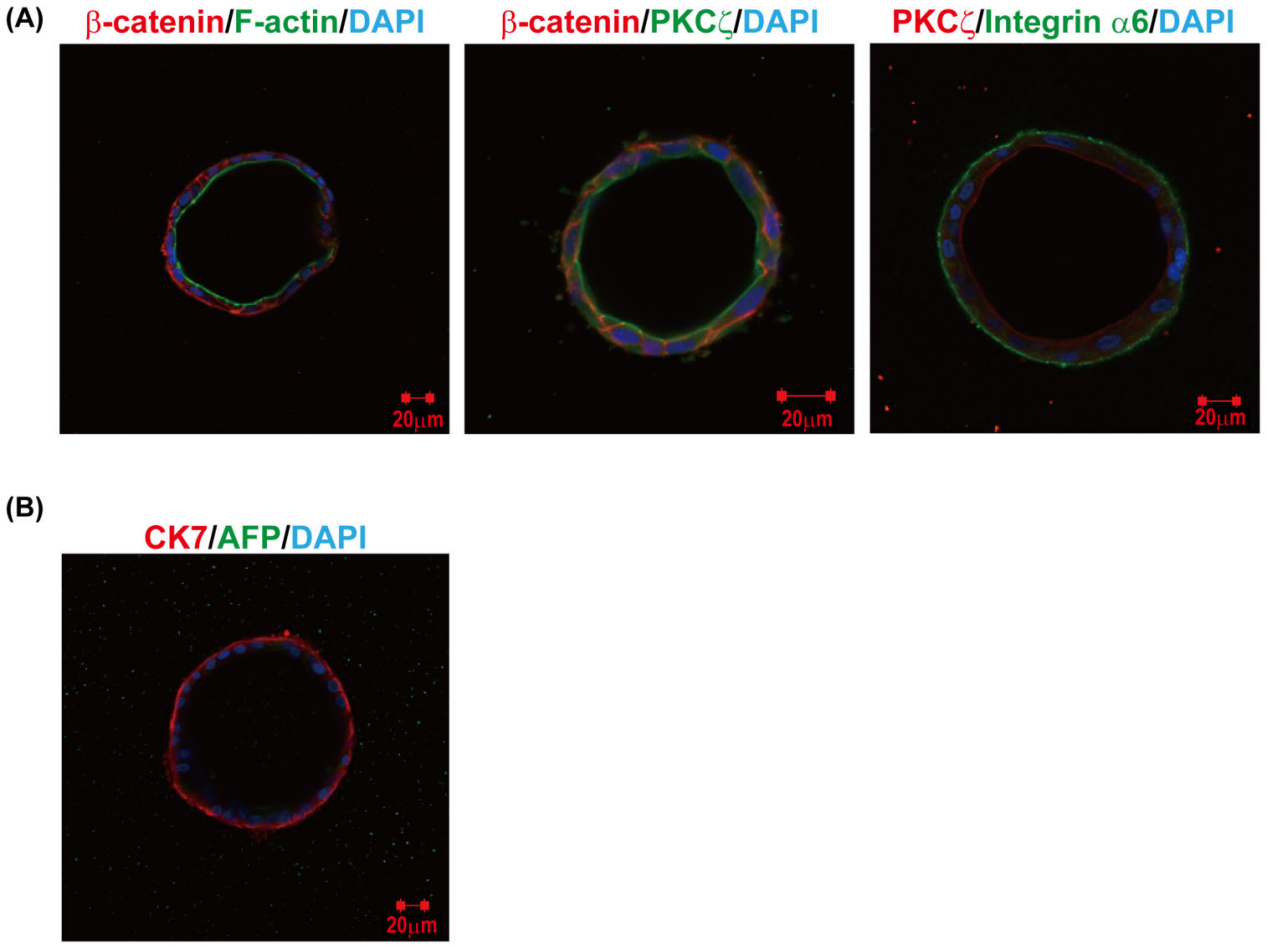
Cholangiocytic cyst formation of human iPS cell-derived HPCs. (**A**) Human iPS cell-derived HPC colonies were trypsinized and cultured in an extracellular matrix gel. After 10–12 days of culture, several numbers of epithelial cysts were formed. Expression of β-catenin, F-actin, integrin α6 and PKCζ was detected in cysts. (**B**) Expression of CK7 in a HPC-derived epithelial cyst. Cells were stained with antibodies against CK7 and AFP. Nuclei were counterstained with DAPI.

It has been reported that cholangiocytic cells form cysts with an epithelial polarity, demonstrating *in vitro* tubulogenesis in an extracellular matrix gel supplemented with cytokines [20]. We therefore examined whether human iPS cell-derived HPCs could form a cholangiocytic structure during 3D-gel culture. Human iPS cell-derived HPC colonies were trypsinized and cultured in an extracellular matrix gel supplemented with EGF, HGF, R-spondin 1, Wnt-3a, A-83-01, and Y-27632. After 12 days of culture, many epithelial cysts were formed. We analyzed the expression of F-actin, protein kinase Cζ (PKCζ), integrin α6 (CD49f) and β-catenin as markers for the apical and basolateral domains [20]. Luminal space surrounded by F-actin bundles and PKCζ was detected, and the expression of integrin α6 and β-catenin was located at the basolateral region (**Figure 8A**). Some of these epithelial cysts expressed CK7 but not expressed hepatocytic marker AFP (**Figure 8B**), suggesting that these cells could form cholangiocyte-like cyst structures with an epithelial polarity. These results indicate that human iPS cell-derived HPCs exhibit a bipotent differentiation ability toward hepatocytes and cholangiocytes.

### Purification and expansion of HPCs derived from human ES cells

Differentiated human ES cells (KhES-3) toward hepatic lineage cells also contained CD13^high^CD133^+^ cells (**Figure S5A**). These human ES cell-derived CD13^high^CD133^+^ fractions formed large colonies on MEFs in H-CFU-C medium supplemented with EGF, HGF, A-83-01, and Y-27632. To analyze whether human ES cell-derived CD13^high^CD133^+^ cells could proliferate in long-term cultures *in vitro*, colonies from human ES cell-derived CD13^high^CD133^+^ cells were trypsinized and replated onto new feeder cells. Similar to the results for human iPS cells, human ES cell-derived CD13^high^CD133^+^ cells continued to proliferate for a long time (at least 1 month). CD13^high^CD133^+^-derived colonies expressed the hepatocytic markers AFP and HNF4α and the endodermal marker HNF3β (**Figure S5B**). A small number of cells in colonies of the 1st culture expressed albumin (**Figure S5C**). The proliferation marker Ki67 and HNF4α were also expressed in human ES cell-derived CD13^high^CD133^+^-derived colonies after the culture passage (**Figure S6**). These cells did not express Oct3/4. The cells had hepatic progenitor-like potential, similar to HPCs from human iPS cell-derived CD13^high^CD133^+^ cells. These results indicate that our methods are useful for expansion of HPCs derived from not only human iPS cells, but also human ES cells.

## Discussion

In the present study, we showed that CD13 and CD133 are specific cell surface markers of HPCs derived from human iPS cells. When human iPS cells were stimulated with suitable cytokines, a small population of cells began to express CD13 and CD133, and could expand in long-term culture. CD13 and CD133 are specific markers of mouse fetal hepatoblasts and adult liver progenitor cells [16], [17], [36]. CD13 is also expressed in human liver cancer stem cells [37]. CD133 is known as an important stem cell marker because many types of cancer stem cells and somatic stem/progenitor cells, such as neural, intestine and hematopoietic stem cells, express this cell surface marker [38], [39], [40], [41]. These results suggest that somatic and cancer stem/progenitor cells share similar cell surface proteins. A population of human iPS cells cultured without cytokines spontaneously expressed a moderate level of CD13. In contrast, CD13^high^CD133^+^ cells were only detected in cultures with appropriate cytokine stimulation. Current protocols for induction of the hepatocytic lineage from human iPS cells have been established by mimicking the events that occur during developmental stages. Such protocols contain 4 steps: (1) induction of endodermal progenitor cells by activin A, (2) hepatocytic specification by basic FGF and BMP-4, (3) differentiation to hepatic stem/progenitor-like cells by HGF, (4) maturation to hepatocytes by OSM. We purified HPCs derived from human iPS cells when these cells were cultured at step (3). It was reported that self-renewing endodermal progenitors derived from human ES cells and iPS cells were established [14]. These cells expressed both CD117 and CXCR4, endodermal progenitor cell markers, and had multipotency to differentiate into pancreatic, hepatic, and intestinal cells. In contrast, the CD13^high^CD133^+^ HPCs in the present study expressed AFP, a primitive hepatocytic marker, but not CXCR4 (**Figure 1C** and data not shown), suggesting that our cells were committed progenitor cells. A previous study reported that N-cadherin is a cell surface marker of hepatic progenitor cells derived from human ES cells stimulated with activin A and basic FGF. However, only a few cells in N-cadherin-positive populations can form large hepatocytic colonies [15]. In contrast, the present study clearly indicates that the usage of CD13 and CD133 in combination with our culture condition is suitable for purification and expansion of HPCs.

A-83-01 and Y-27632 were important for the expansion of human iPS cell-derived HPCs. Human ES and iPS cells are not able to survive as single cells because activation of the Rho-Rock signal induces blebbing and apoptosis of these cells [42]. Survival and expansion of hepatic progenitor cells derived from early fetal and adult livers are also induced by ROCK inhibition [17], [36], [43]. Further studies are needed to clarify whether inhibition of ROCK acts in anti-apoptosis and/or proliferation of human iPS cell-derived HPCs. TGFβ and ALK signaling pathways are involved in regeneration and epithelial-mesenchymal transition of liver cells [44]. In this study, we found that inhibition of the ALK signal is required for colony formation of HPCs. The molecular mechanism of the ALK inhibitor regulating human iPS cell-derived HPCs remains unknown. However, activation of TGFβ-ALK signals, such as phosphorylation of Smad family proteins, might cause cell cycle arrest or epithelial-mesenchymal transition of HPCs.

It has been reported that iPS cells generated by retroviral vectors re-express Yamanaka factors in culture during differentiation of several cell types [18], [45]. In our culture system, HPCs derived from human iPS cells could proliferate over a period of 1 month, while maintaining a bipotent differentiation ability. Interestingly, there was no re-activation of exogenous Yamanaka factors (Oct3/4, Klf4, Sox2, and c-Myc) during expansion of these cells, suggesting that exogenous genes (particularly c-Myc) were not involved in the proliferative ability of our HPCs (data not shown). Cell cycle-dependent kinases and their inhibitors are important regulators for the cell cycle and long-term proliferation of stem/progenitor cells. During *in vitro* expansion of mouse hepatocytic cells, up-regulation of p19^ARF^ (p14 ^ARF^ in human cells) induces cell cycle arrest and senescence [46]. In contrast, activation of p16^Ink4a^ and p14^ARF^ was detected during the long-term proliferation of human iPS cell-derived HPCs (data not shown). These results suggest that there may be unknown mechanisms regulating the cell cycle of these cells *in vitro*.

Analyses of human hepatic progenitor cells are difficult because of the shortage of human fetal tissue samples and culture systems. In this regard, our human HPCs derived from iPS cells might be useful for the analysis of human hepatic cell development. In addition, mature hepatocytes can barely maintain a proliferative ability following cryopreservation, whereas our human iPS cell-derived HPCs have a highly proliferate ability even after cryopreservation. Thus, the *in vitro* expansion system presented here may contribute to regenerative therapies of liver diseases using functional human hepatic progenitor cells and hepatocytes.

## Supporting Information

Figure S1 **Expression of cell surface markers in human iPS cell-derived hepatic lineage cells.** (**A**) Expression of hepatic progenitor marker CD49f in CD13^high^CD133^+^ cells. Human iPS cells were stimulated with cytokines and stained with suitable antibodies. CD13^high^CD133^+^ cells slightly expressed CD49f. (**B**) Expression of progenitor cell markers CD56 and CD34 in CD13^high^CD133^+^ cells.
(TIF)

Figure S2 **Differentiation of human iPS cells into hepatic lineage cells.** (**A**) Expressions of AFP (red) and HNF4α (green) in differentiated iPS cells at step (3) in Figure 1. The four fields of view are shown. Many cells have differentiated into AFP- and HNF4α-positive hepatocytic cells, although several cell clusters have not differentiated (arrowheads). (**B**) Expressions of AFP (red) and Oct3/4 (green) in differentiated iPS cells at step (3). Oct3/4 is not expressed in the AFP-positive cells. (**C**) Expressions of AFP (green) and T (red) in differentiated iPS cells at step (3). T is not expressed in the AFP-positive cells. (**A–C**) Nuclei were stained with DAPI (blue).
(TIF)

Figure S3 **Long-term proliferation of human iPS cell-derived HPCs.** (**A**) Representative image of colonies of long-term proliferative human iPS cell-derived HPCs. The colonies were passaged six times and cultured for a total of 90 days after the first sorting. (**B**) Expressions of hepatocytic marker genes in the long-term culture. The colonies were cultured as described for (A) and fixed with 4% PFA. AFP (red) and HNF4α (green) were stained with suitable antibodies. (**C**) After 12 days of culture with cytokines, CD13^high^CD133^+^ cells were sorted onto MEFs. After two passages, the 3rd cultured-cells were trypsinized and stained with antibodies against CD13 and CD133. CD13^+^ (red) and CD13^−^ (blue) cells were purified and serially cultured (4th and 5th cultured-cells). 11d culture: 11-day culture. (**D**) Expansion of CD13^+^ and CD13^−^ cells after long-term culture. As shown in (C), CD13^+^ (red) and CD13^−^ (blue) cells in the 5th-cultured cells were purified and cultured for 9 days on MEFs. The results are represented as the mean colony counts ± SD (duplicate samples).
(TIF)

Figure S4 **Differentiation of human iPS cell-derived HPCs toward mature hepatocytic cells.** (**A**) Schematic diagram of the experimental procedure. HPCs in the 3rd culture were dissociated with 0.05% trypsin-EDTA. Spheroids derived from HPCs were formed using hanging drop culture. (**B**) Expression of albumin in HPCs matured by cell-cell interactions. (**C**) Albumin secretion by human iPS cell-derived HPCs is identified after 3 days of culture in medium by enzyme-linked immunosorbent assays.
(TIF)

Figure S5 **Purification of human ES cell-derived HPCs.** (**A**) Expressions of CD13 and CD133, cell surface markers of hepatic progenitor cells, in human ES cells cultured with or without cytokines. After 12 days of culture, the cells were stained with antibodies against CD13 and CD133, and then analyzed by flow cytometry. (**B**) Expressions of hepatocytic and cholangiocytic markers during *in vitro* expansion of human ES cell-derived HPCs. Colonies derived from CD13^high^CD133^+^ cells were cultured on MEFs. The expressions of several liver markers are detected in the 1st and 2nd cultures. An endodermal marker (HNF3β), hepatocytic markers (AFP and HNF4α), and a cholangiocytic marker (CK7) were stained with specific antibodies. (**C**) Expression of albumin in colonies derived from human ES cell-derived CD13^high^CD133^+^ cells. Albumin is detected in several colonies in the 1st culture.
(TIF)

Figure S6 **Proliferative ability of human ES cell-derived CD13^high^CD133^+^ cells.** Expressions of a pluripotency marker (Oct3/4) and a proliferation marker (Ki67) are observed in colonies derived from human ES cell-derived CD13^high^CD133^+^ cells. Ki67-expressing proliferative cells express HNF4α in the 2nd culture. These cells do not express Oct3/4. Nuclei were counterstained with DAPI.
(TIF)

Table S1 **List of antibodies used for immunostaining and flow cytometry experiments.**
(DOCX)

Table S2 **Lists of PCR primers for detection of human gene expression.** Afp, α-fetoprotein alpha; COMT, catechol-O-methyltransferase; CXCR4, chemokine (C-X-C motif) receptor 4; CYP, cytochrome P450; EPHX1, epoxide hydrolase 1, microsomal (xenobiotic); FMO5, flavin containing monooxygenase 5; GSC, goosecoid homeobox; hHex, hematopoietically expressed homeobox; HNF, hepatocyte nuclear factor; HPRT1, hypoxanthine phosphoribosyltransferase 1; MAO, monoamine oxidase; MIXL1, Mix paired-like homeobox; ONECUT1, one cut homeobox 1; Sox17, SRY-box containing gene 17; SULT1A1, sulfotransferase family, cytosolic, 1A, phenol-preferring, member1.
(DOCX)
