# Supplementary figures and images for: An In Vitro Expansion System for Generation of Human iPS Cell-Derived Hepatic Progenitor-Like Cells Exhibiting a Bipotent Differentiation Potential
Source: PLoS One. 2013 Jul 25;8(7):e67541. doi: 10.1371/journal.pone.0067541 (PMC3723819; doi:10.1371/journal.pone.0067541)

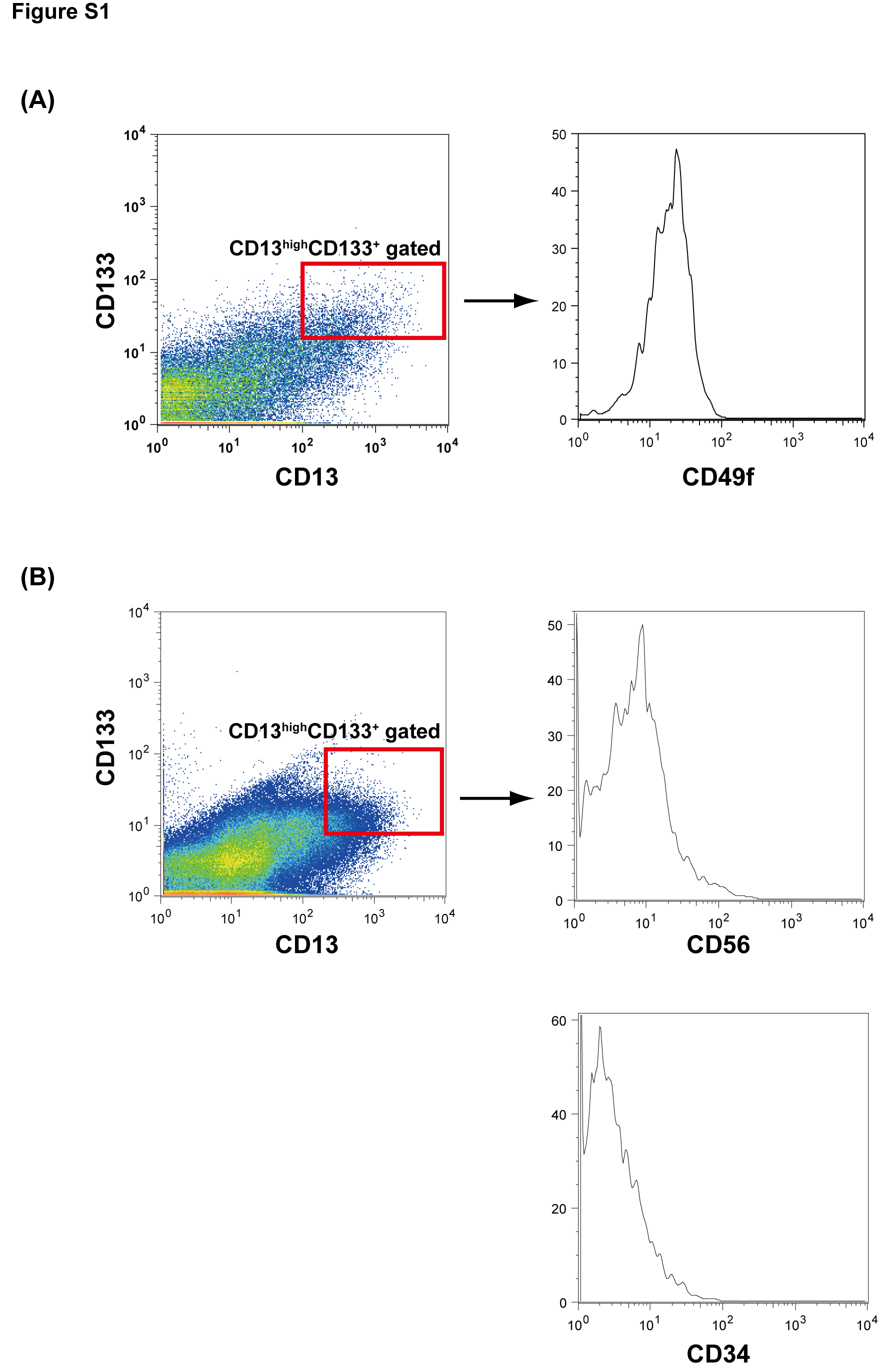

Supplement: Figure S1 — Expression of cell surface markers in human iPS cell-derived hepatic lineage cells. (A) Expression of hepatic progenitor marker CD49f in CD13highCD133+ cells. Human iPS cells were stimulated with cytokines and stained with suitable antibodies. CD13highCD133+ cells slightly expressed CD49f. (B) Expression of progenitor cell markers CD56 and CD34 in CD13highCD133+ cells. (TIF) [file pone.0067541.s001.tif]

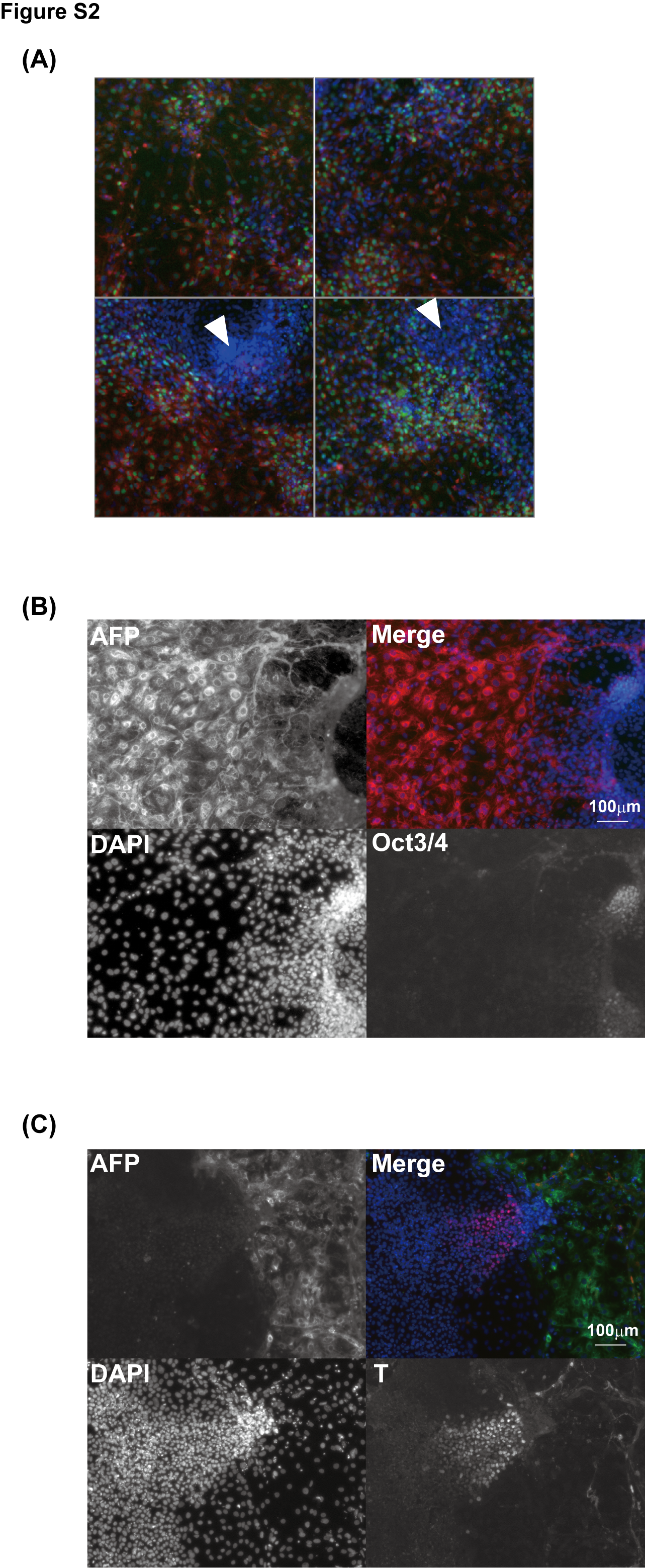

Supplement: Figure S2 — Differentiation of human iPS cells into hepatic lineage cells. (A) Expressions of AFP (red) and HNF4α (green) in differentiated iPS cells at step (3) in Figure 1. The four fields of view are shown. Many cells have differentiated into AFP- and HNF4α-positive hepatocytic cells, although several cell clusters have not differentiated (arrowheads). (B) Expressions of AFP (red) and Oct3/4 (green) in differentiated iPS cells at step (3). Oct3/4 is not expressed in the AFP-positive cells. (C) Expressions of AFP (green) and T (red) in differentiated iPS cells at step (3). T is not expressed in the AFP-positive cells. (A–C) Nuclei were stained with DAPI (blue). (TIF) [file pone.0067541.s002.tif]

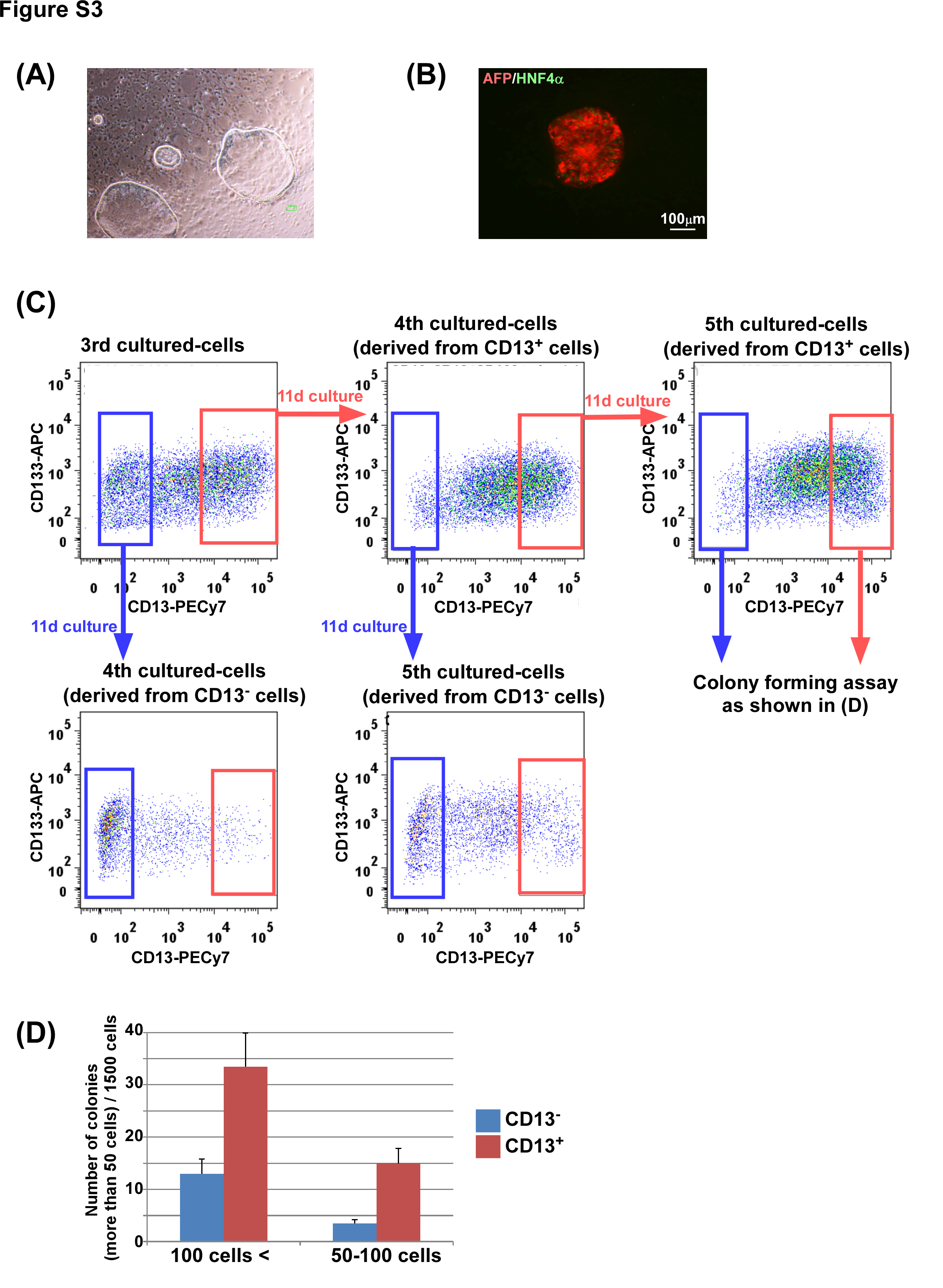

Supplement: Figure S3 — Long-term proliferation of human iPS cell-derived HPCs. (A) Representative image of colonies of long-term proliferative human iPS cell-derived HPCs. The colonies were passaged six times and cultured for a total of 90 days after the first sorting. (B) Expressions of hepatocytic marker genes in the long-term culture. The colonies were cultured as described for (A) and fixed with 4% PFA. AFP (red) and HNF4α (green) were stained with suitable antibodies. (C) After 12 days of culture with cytokines, CD13highCD133+ cells were sorted onto MEFs. After two passages, the 3rd cultured-cells were trypsinized and stained with antibodies against CD13 and CD133. CD13+ (red) and CD13− (blue) cells were purified and serially cultured (4th and 5th cultured-cells). 11d culture: 11-day culture. (D) Expansion of CD13+ and CD13− cells after long-term culture. As shown in (C), CD13+ (red) and CD13− (blue) cells in the 5th-cultured cells were purified and cultured for 9 days on MEFs. The results are represented as the mean colony counts ± SD (duplicate samples). (TIF) [file pone.0067541.s003.tif]

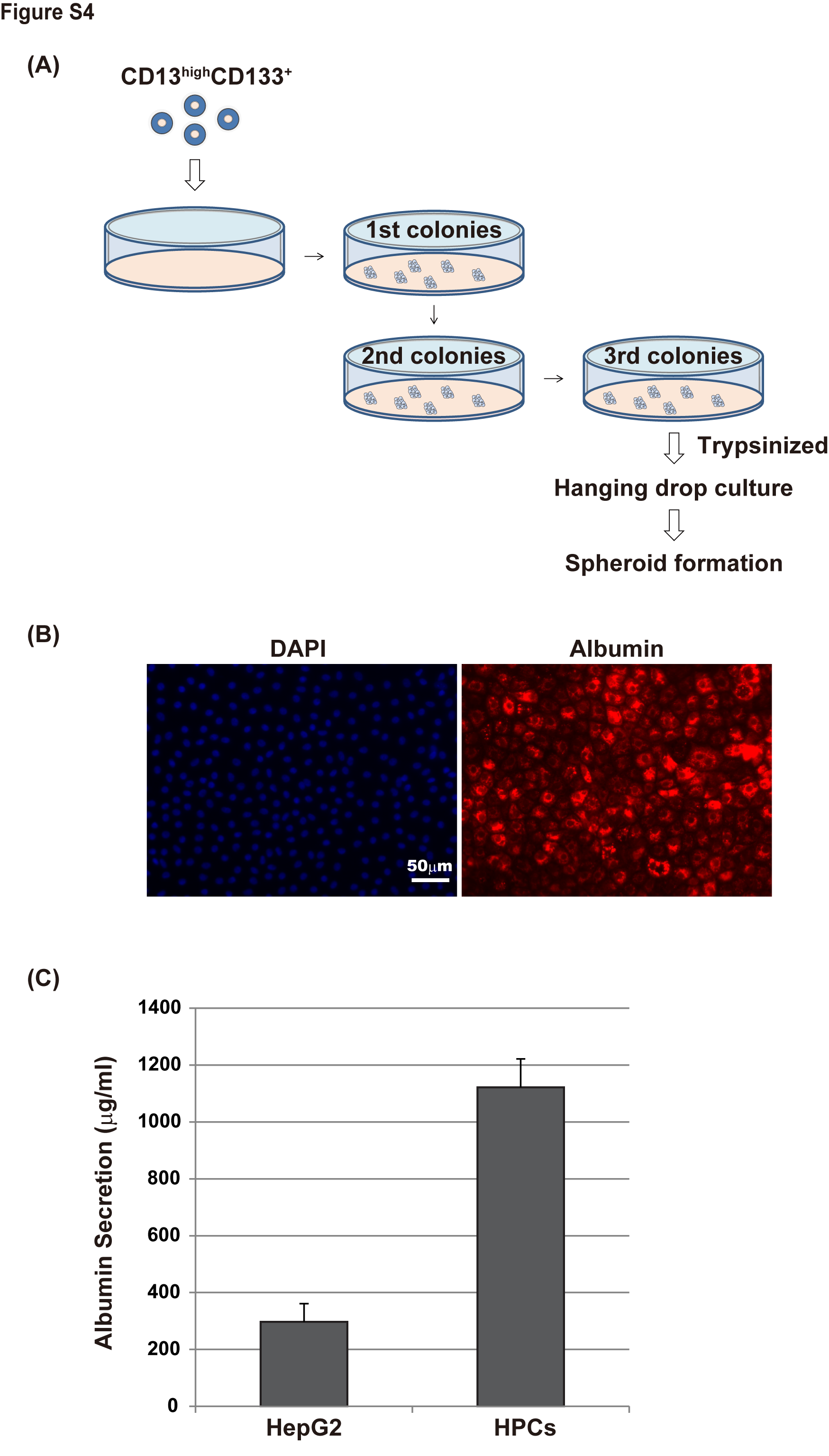

Supplement: Figure S4 — Differentiation of human iPS cell-derived HPCs toward mature hepatocytic cells. (A) Schematic diagram of the experimental procedure. HPCs in the 3rd culture were dissociated with 0.05% trypsin-EDTA. Spheroids derived from HPCs were formed using hanging drop culture. (B) Expression of albumin in HPCs matured by cell-cell interactions. (C) Albumin secretion by human iPS cell-derived HPCs is identified after 3 days of culture in medium by enzyme-linked immunosorbent assays. (TIF) [file pone.0067541.s004.tif]

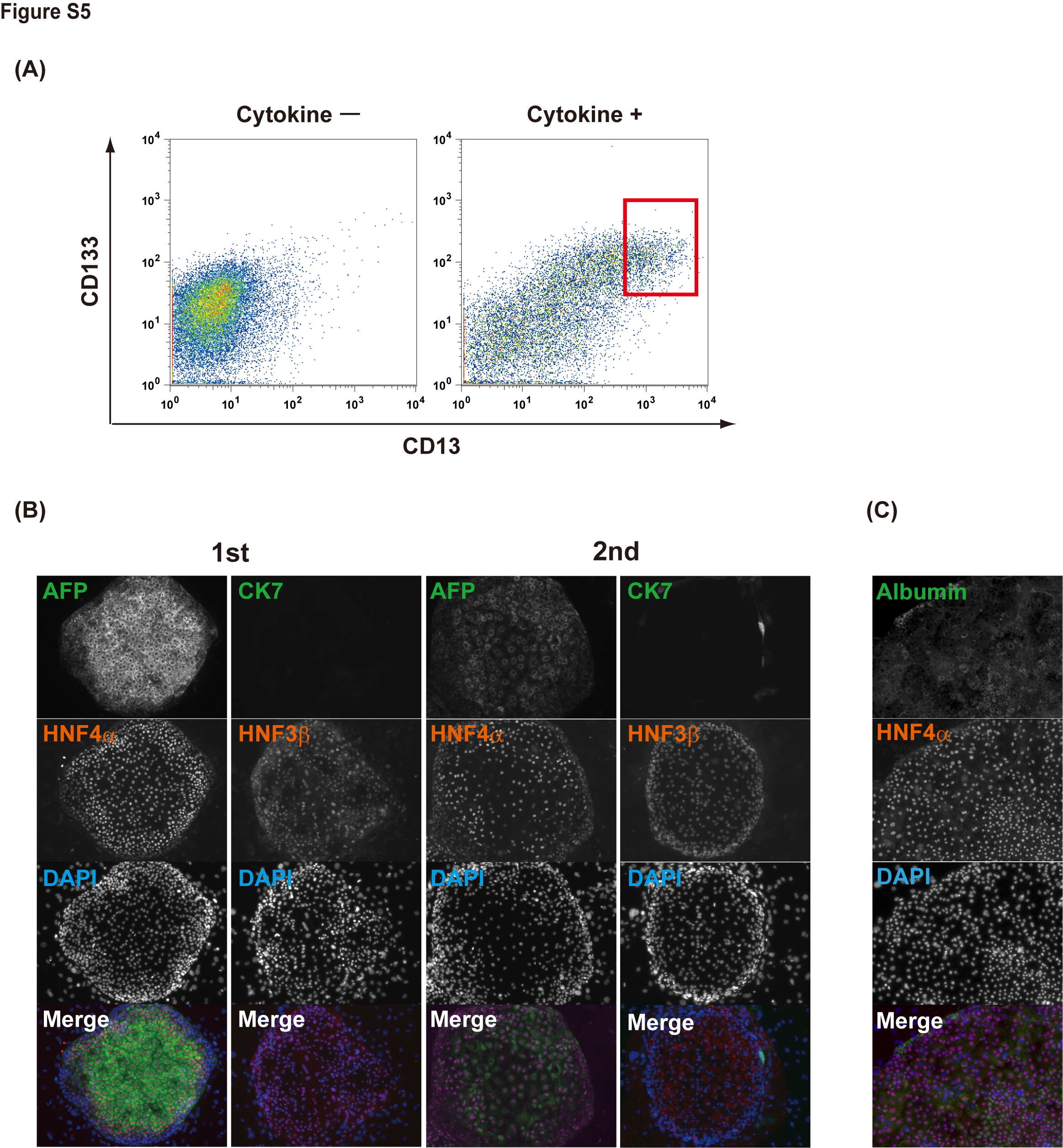

Supplement: Figure S5 — Purification of human ES cell-derived HPCs. (A) Expressions of CD13 and CD133, cell surface markers of hepatic progenitor cells, in human ES cells cultured with or without cytokines. After 12 days of culture, the cells were stained with antibodies against CD13 and CD133, and then analyzed by flow cytometry. (B) Expressions of hepatocytic and cholangiocytic markers during in vitro expansion of human ES cell-derived HPCs. Colonies derived from CD13highCD133+ cells were cultured on MEFs. The expressions of several liver markers are detected in the 1st and 2nd cultures. An endodermal marker (HNF3β), hepatocytic markers (AFP and HNF4α), and a cholangiocytic marker (CK7) were stained with specific antibodies. (C) Expression of albumin in colonies derived from human ES cell-derived CD13highCD133+ cells. Albumin is detected in several colonies in the 1st culture. (TIF) [file pone.0067541.s005.tif]

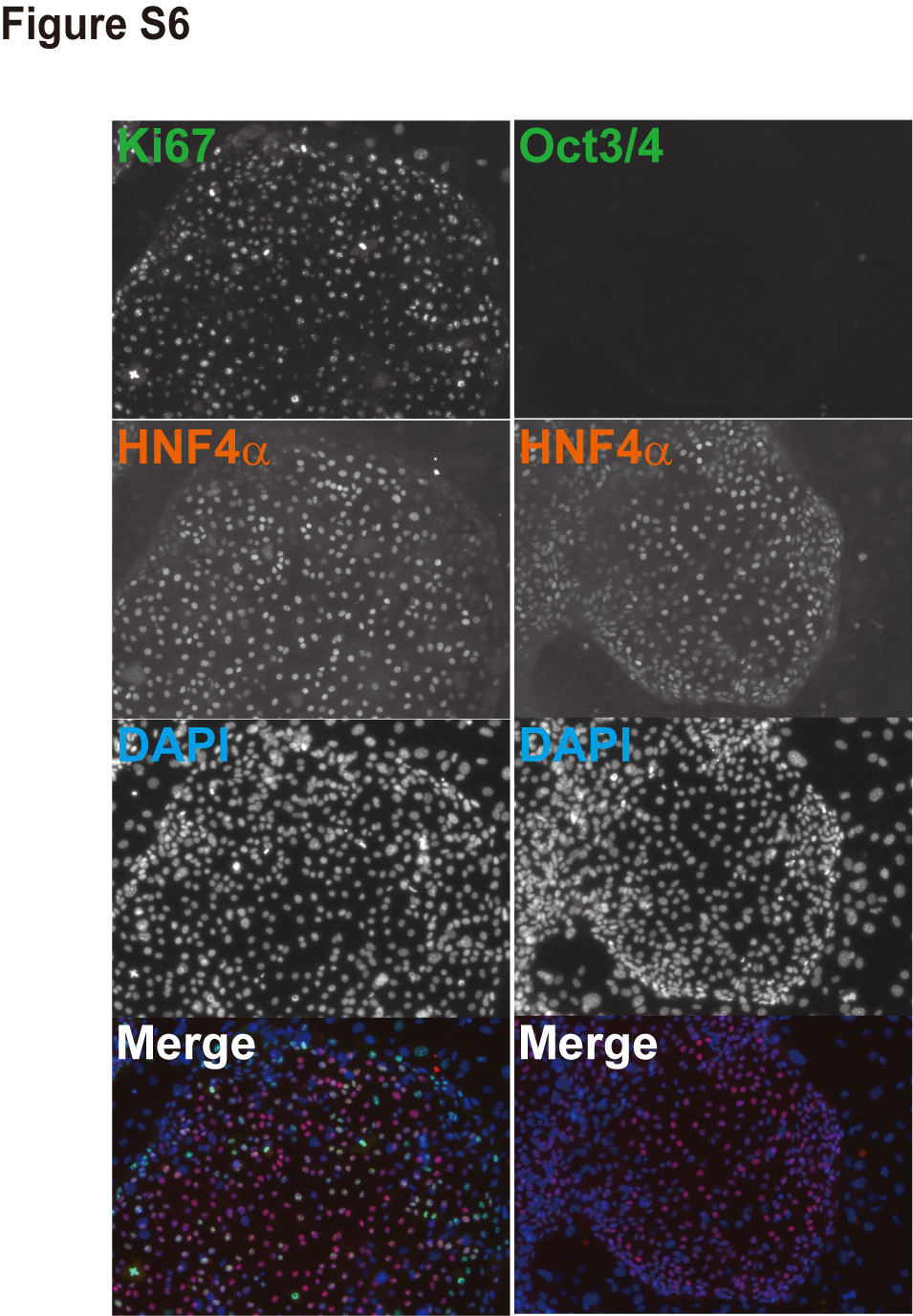

Supplement: Figure S6 — Proliferative ability of human ES cell-derived CD13highCD133+ cells. Expressions of a pluripotency marker (Oct3/4) and a proliferation marker (Ki67) are observed in colonies derived from human ES cell-derived CD13highCD133+ cells. Ki67-expressing proliferative cells express HNF4α in the 2nd culture. These cells do not express Oct3/4. Nuclei were counterstained with DAPI. (TIF) [file pone.0067541.s006.tif]
