# Supplementary material for: An In Vitro Expansion System for Generation of Human iPS Cell-Derived Hepatic Progenitor-Like Cells Exhibiting a Bipotent Differentiation Potential
Source: PLoS One. 2013 Jul 25;8(7):e67541. doi: 10.1371/journal.pone.0067541 (PMC3723819; doi:10.1371/journal.pone.0067541)
Supplement: Table S1 — List of antibodies used for immunostaining and flow cytometry experiments. (DOCX) [file pone.0067541.s007.docx]

**Table S1.** List of antibodies used for immunostaining and flow cytometry experiments

| **Primary antibodies for flow cytometry** | **Clone** | **Source** | **Catalog number** |  |  |
| --- | --- | --- | --- | --- | --- |
| CD13-PE | WM15 | BD Pharmingen | 555394 |  |  |
| CD13-PE-Cy7 | WM15 | BD Pharmingen | 561599 |  |  |
| CD34-FITC | 581/CD34 | BD Pharmingen | 555821 |  |  |
| CD44-FITC | IM7 | eBioscience | 11-0441-82 |  |  |
| CD49f (Integrin α6)-FITC | GoH3 | BD Pharmingen | 555735 |  |  |
| CD56-PE-Cy7 | B159 | BD Pharmingen | 557747 |  |  |
| CD117-APC | YB5.B8 | BD Pharmingen | 550412 |  |  |
| CD133/1-APC | AC133 | Miltenyi Biotec | 130-090-826 |  |  |
| CD184 (CXCR4)-PE | 12G5 | eBioscience | 12-9999-71 |  |  |
| CD326-Alexa Fluor 488 | 9C4 | Biolegend | 324210 |  |  |
|  |  |  |  |  | |
| **Primary antibodies for immunostaining** | **Dilution** | **Source** | **Catalog number** |  |  |
| α-fetoprotein (AFP) (C3) | 1/600 | Sigma | A8452 |  |  |
| α-fetoprotein (AFP) (C3) | 1/600 | Dako | A0008 |  |  |
| Albumin | 1/1000 | Dako | A000102 |  |  |
| β-catenin | 1/1000 | BD Pharmingen | 610154 |  |  |
| Brachyury (T) (C-19) | 1/250 | Santa Cruz | sc-17745 |  |  |
| Cytokeratin-7 (CK7)  (OV-TL 12/30) | 1/1000 | Dako | M7018 |  |  |
| F-actin  (Acti-stain 488 phalloidin) | 1/150 | Cytoskeleton, Inc. | PHDG1 |  |  |
| HNF4α (C-19) | 1/600 | Santa Cruz | sc-6556 |  |  |
| HNF3β (M-20) | 1/300 | Santa Cruz | sc-6554 |  |  |
| Integrin α6 (CD49f) (GoH3) | 1/500 | BD Pharmingen | 555734 |  |  |
| Ki67 | 1/500 | Abcam | ab15580 |  |  |
| Oct-3/4 (C-10) | 1/100 | Santa Cruz | sc-5279 |  |  |
| Protein kinase ζ (C-20) | 1/500 | Santa Cruz | sc-216 |  |  |
|  |  |  |  |  |  |
| **Secondary antibodies** | **Dilution** | **Source** | **Catalog number** |  |  |
| anti-mouse/Alexa Fluor 488 | 1/1000 | Invitrogen | A21202 |  |  |
| anti-rabbit/Alexa Fluor 488 | 1/1000 | Invitrogen | A21206 |  |  |
| anti-rat/Alexa Fluor 488 | 1/1000 | Invitrogen | A21208 |  |  |
| anti-goat/Alexa Fluor 488 | 1/1000 | Invitrogen | A11055 |  |  |
| anti-goat/Alexa Fluor 546 | 1/1000 | Invitrogen | A11056 |  |  |
| anti-mouse/Alexa Fluor 555 | 1/1000 | Invitrogen | A31570 |  |  |
| anti-rabbit/Alexa Fluor 555 | 1/1000 | Invitrogen | A31572 |  |  |
| anti-rabbit/Alexa Fluor 568 | 1/1000 | Invitrogen | A10042 |  |  |
