# Supplementary material for: An In Vitro Expansion System for Generation of Human iPS Cell-Derived Hepatic Progenitor-Like Cells Exhibiting a Bipotent Differentiation Potential
Source: PLoS One. 2013 Jul 25;8(7):e67541. doi: 10.1371/journal.pone.0067541 (PMC3723819; doi:10.1371/journal.pone.0067541)
Supplement: Table S2 — Lists of PCR primers for detection of human gene expression. Afp, α-fetoprotein alpha; COMT, catechol-O-methyltransferase; CXCR4, chemokine (C-X-C motif) receptor 4; CYP, cytochrome P450; EPHX1, epoxide hydrolase 1, microsomal (xenobiotic); FMO5, flavin containing monooxygenase 5; GSC, goosecoid homeobox; hHex, hematopoietically expressed homeobox; HNF, hepatocyte nuclear factor; HPRT1, hypoxanthine phosphoribosyltransferase 1; MAO, monoamine oxidase; MIXL1, Mix paired-like homeobox; ONECUT1, one cut homeobox 1; Sox17, SRY-box containing gene 17; SULT1A1, sulfotransferase family, cytosolic, 1A, phenol-preferring, member1. (DOCX) [file pone.0067541.s008.docx]

**Table S2.** PCR primers for detection of human gene expression

| **Human genes** | **Forward primer (5’–3’)** | **Reverse primer (5’–3’)** | **Probe number** |
| --- | --- | --- | --- |
| *AFP* | tgtactgcagagataagtttagctgac | tccttgtaagtggcttcttgaac | 61 |
| *COMT* | tgcacacactaccaatcgttc | gcctgggcccttgtagat | 10 |
| *CXCR4* | ggtggtctatgttggcgtct | actgacgttggcaaagatga | 18 |
| *CYP3A4* | gatggctctcatcccagactt | agtccatgtgaatgggttcc | [2](https://qpcr.probefinder.com/showsequence.jsp?seqNo=1231623940) |
| *CYP3A7* | caaaagactctgagacccacaa | agccagcaaaaataaagataattga | [50](https://qpcr.probefinder.com/showsequence.jsp?seqNo=417983544) |
| *CYP7A1* | gcaggcacctgtagtcttagc | cggagacgggatctcactaa | 64 |
| *EPHX1* | gatgacccagaagcatgagc | gcgtgtgcaatagctcaaaa | 8 |
| *FMO5* | attagccaaacagccaagca | acacgattcaggatccaagc | 73 |
| *GSC* | cctccgcgaggagaaagt | cgttctccgactcctctgat | [29](https://qpcr.probefinder.com/showsequence.jsp?seqNo=1930932210) |
| *hHex* | cggacggtgaacgactaca | agaaggggctccagagtagag | 61 |
| *HNF3β* | cccaatcttgacacggtga | aaataaagcacgcagaaacca | 85 |
| *HNF4α* | gagatccatggtgttcaagga | gtgccgagggacaatgtagt | 68 |
| *HPRT1* | tgaccttgatttattttgcatacc | cgagcaagacgttcagtcct | [73](https://qpcr.probefinder.com/showsequence.jsp?seqNo=1799438811) |
| *MAOA* | attaagtgcatgatgtattacaaggag | tggagcatcttcatcttcaatg | 5 |
| *MAOB* | ctggcagtcagaaccagagtc | gagggcaaatgtctctccaa | 9 |
| *MIXL1* | ctgaggagccatgactgaca | gcatggaagtcagaaaggaca | 14 |
| *ONECUT1* | cctggagcaaactcaaatcc | ttctttccttttgcatgctg | 88 |
| *Sox17* | acgccgagttgagcaaga | tctgcctcctccacgaag | [61](https://qpcr.probefinder.com/showsequence.jsp?seqNo=1363943168) |
| *SULT1A1* | aagtgtcctacggatcctggt | tctcccttttcgggttctc | 24 |
| *SULT1A2* | gacctgggaaagcttcctg | tggtaccaggacccatagga | 19 |
| *T* | gctgtgacaggtacccaacc | ggagaattgttccgatgagc | 23 |
